# Prenatal Exposure to NO_2_ and Ultrasound Measures of Fetal Growth in the Spanish INMA Cohort

**DOI:** 10.1289/ehp.1409423

**Published:** 2015-06-26

**Authors:** Carmen Iñiguez, Ana Esplugues, Jordi Sunyer, Mikel Basterrechea, Ana Fernández-Somoano, Olga Costa, Marisa Estarlich, Inmaculada Aguilera, Aitana Lertxundi, Adonina Tardón, Mònica Guxens, Mario Murcia, Maria-Jose Lopez-Espinosa, Ferran Ballester, on behalf of the INMA Project

**Affiliations:** 1 Epidemiology and Environmental Health Joint Research Unit, FISABIO–Universitat Jaume I–Universitat de València, Valencia, Spain; 2 Spanish Consortium for Research on Epidemiology and Public Health (CIBERESP), Madrid, Spain; 3 Center for Research in Environmental Epidemiology (CREAL), Barcelona, Spain; 4 Hospital del Mar Research Institute (IMIM), Barcelona, Spain; 5 Pompeu Fabra University, Barcelona, Spain; 6 Public Health Division, Basque Government, Spain; 7 Health Research Institute, Biodonostia, San Sebastián, Spain; 8 University of Oviedo, Oviedo, Spain; 9 Swiss Tropical and Public Health Institute, Basel, Switzerland; 10 University of Basel, Basel, Switzerland; 11 University of the Basque Country (UPV/EHU), Bizkaia, Spain; 12 Department of Child and Adolescent Psychiatry/Psychology, Erasmus University Medical Centre–Sophia Children’s Hospital, Rotterdam, the Netherlands

## Abstract

**Background:**

Air pollution exposure during pregnancy has been associated with impaired fetal growth. However, few studies have measured fetal biometry longitudinally, remaining unclear as to whether there are windows of special vulnerability.

**Objective:**

The aim was to investigate the impact of nitrogen dioxide (NO_2_) exposure on fetal and neonatal biometry in the Spanish INMA study.

**Methods:**

Biparietal diameter (BPD), femur length (FL), abdominal circumference (AC), and estimated fetal weight (EFW) were evaluated for up to 2,478 fetuses in each trimester of pregnancy. Size at 12, 20, and 34 weeks of gestation and growth between these points, as well as anthropometry at birth, were assessed by SD scores derived using cohort-specific growth curves. Temporally adjusted land-use regression was used to estimate exposure to NO_2_ at home addresses for up to 2,415 fetuses. Associations were investigated by linear regression in each cohort and subsequent meta-analysis.

**Results:**

A 10-μg/m^3^ increase in average exposure to NO_2_ during weeks 0–12 was associated with reduced growth at weeks 0–12 in AC (–2.1%; 95% CI: –3.7, –0.6) and EFW (–1.6%; 95% CI: –3.0, –0.3). The same exposure was inversely associated with reduced growth at weeks 20–34 in BPD (–2.6%; 95% CI: –3.9, –1.2), AC (–1.8%; 95% CI: –3.3, –0.2), and EFW (–2.1%; 95% CI: –3.7, –0.2). A less consistent pattern of association was observed for FL. The negative association of this exposure with BPD and EFW was significantly stronger in smoking versus nonsmoking mothers.

**Conclusions:**

Maternal exposure to NO_2_ in early pregnancy was associated with reduced fetal growth based on ultrasound measures of growth during pregnancy and measures of size at birth.

**Citation:**

Iñiguez C, Esplugues A, Sunyer J, Basterrechea M, Fernández-Somoano A, Costa O, Estarlich M, Aguilera I, Lertxundi A, Tardón A, Guxens M, Murcia M, Lopez-Espinosa MJ, Ballester F, on behalf of the INMA Project. 2016. Prenatal exposure to NO_2_ and ultrasound measures of fetal growth in the Spanish INMA Cohort. Environ Health Perspect 124:235–242; http://dx.doi.org/10.1289/ehp.1409423

## Introduction

Fetal development is a global public health concern because growth *in utero* is a good indicator of perinatal and postnatal health (Gluckman et al. 2008; Kramer 2003). Its vulnerability to air toxicants is relevant because air pollution, particularly traffic-related, is a well-known, ubiquitous, and potentially modifiable environmental risk factor (Perez et al. 2013). The study of the effects of air pollution on fetal growth may help to outline the first step on the causal pathway to the association between prenatal air pollution exposure and adverse health effects later in life, such as childhood obesity (Rundle et al. 2012), cardiovascular disease (Kelishadi and Poursafa 2014), respiratory morbidity (Patel et al. 2011), or neurological disorders (Guxens et al. 2012a).

Previous literature has provided suggestive evidence on the adverse effects of air pollution on small for gestational age (SGA), low birth weight (LBW), and other markers of impaired fetal growth assessed at birth (Estarlich et al. 2011; Srám et al. 2005; Stieb et al. 2012), even at pollution levels authorized by current legislation (Pedersen et al. 2013). Nevertheless, because an assessment at birth does not fully capture the timing of changes over the course of the pregnancy, results from the majority of previous studies are unable to add evidence on the age at which fetal growth failure begins or on transient effects that may be compensated for in the remaining intrauterine life.

The timing of exposure to ambient toxicants could, in consequence, play a key role in the identification of critical exposure windows within pregnancy that may help to disentangle the underlying mechanisms (Slama et al. 2008a). Exposures during early pregnancy may result in disruption of placental growth and functioning, leading to current and later impaired fetal growth (van den Hooven et al. 2012a), whereas exposures during later pregnancy could induce changes in plasma viscosity and artery vasoconstriction, thereby influencing in turn maternal–placental exchanges and hence interfering with the period of increased rates of nutrient requirements (Slama et al. 2008a). In this respect, although adverse associations have been reported more frequently in the first and third trimesters (Ritz and Wilhelm 2008), evidence of exposure effects during specific prenatal periods is still inconclusive (Stieb et al. 2012).

To date only six studies, five of them included in a recent review (Smarr et al. 2013), have estimated the impact of prenatal air pollution exposure on fetal biometry measured via ultrasounds (Aguilera et al. 2010; Hansen et al. 2008; Iñiguez et al. 2012; Ritz et al. 2014; Slama et al. 2009; van den Hooven et al. 2012b). Sample size, exposure windows, and time at ultrasound examination differed among the studies, leading to heterogeneity in reported associations.

The INfancia y Medio Ambiente (INMA)—Childhood and Environment Study—is a network of several population-based birth cohorts in Spain established to evaluate the role of the environment on fetal and childhood health (Guxens et al. 2012b). Two of the six above-mentioned studies were conducted in the two most urban cohorts of INMA, the cohorts of Sabadell and Valencia, respectively (Iñiguez et al. 2012; Aguilera et al. 2010). That none of them found any clear relationship in early pregnancy may reflect the sample size or the exposure variability required to detect an association at that stage of gestation. To increase the statistical power and extend the study to less-exposed populations, we conducted a joint analysis aimed at evaluating the association between prenatal exposure to traffic-related air pollution and fetal biometry at different stages of pregnancy.

## Methods

*Population and study design*. This study was based on the four *de novo* INMA cohorts sited in Asturias, Gipuzkoa, Sabadell, and Valencia (Guxens et al. 2012b). Recruitment took place between 2003 and 2008. A total of 2,644 eligible women (age at least 16 years, at 10–13 weeks of gestation, with a singleton pregnancy, non-assisted conception, and no communication handicap) agreed to participate and signed informed consent forms. After excluding women who withdrew, were lost to follow-up, or underwent induced or spontaneous abortions or fetal deaths, or without at least two valid ultrasounds, the sample consisted of 2,496 pregnant women. The study was approved by the Hospital Ethics Committees in the participating regions.

*Fetal ultrasonography*. Ultrasound scans (Voluson 730 Pro and 730 Expert; Siemens Sienna) were scheduled at 12, 20, and 34 weeks of gestation and performed by obstetricians specialized in conducting this type of examinations at the respective hospitals. We had access to the records of any other ultrasound scan performed on the women during their pregnancy, which allowed us to obtain from two to eight valid ultrasounds per woman between 7 and 42 weeks of gestation. The characteristics examined were biparietal diameter (BPD), femur length (FL), abdominal circumference (AC), and estimated fetal weight (EFW) (Hadlock et al. 1985). An early crown–rump length (CRL) measurement was used for pregnancy dating. Gestational age was established using CRL when the difference with the age based on the self-reported last menstrual period (LMP) was ≥ 7 days. Women with a difference of > 3 weeks (*n* = 18) were excluded to avoid a possible bias. Data outside the range mean ± 4 SD for each gestational age (*n* = 5, 8, and 8 for AC, FL, and BPD, respectively) were also eliminated to avoid the influence of extreme values. In all, 2,478 women provided information for fetal growth modeling.

Linear mixed models (Pinheiro and Bates 2000) were used separately in each cohort to obtain a growth curve for each parameter. Models were adjusted for constitutional factors known to affect fetal growth: maternal age, height, parity, country of origin (as proxy of ethnicity), prepregnancy weight, father’s height, and fetal sex.

In accordance with these customized models, unconditional SD scores at 12, 20, and 34 weeks of gestation and conditional SD scores for 12–20 and 20–34 weeks of gestation were calculated. An unconditional SD score at a certain point describes the size at this time, and an SD score at a certain time point conditioned by the value raised in a previous moment describes the growth experienced in the respective time interval (Gurrin et al. 2001).

To prevent the increase of random error due to small deviations from the scheduled times, we calculated SD scores at a particular time using the prediction (by the corresponding fetal curve) at this particular time point conditioned to the nearest measure. Detailed information about fetal growth modeling may be found in Supplemental Material, “Fetal growth curves and calculation of SD scores” and Figures S1–S5.

*Neonatal outcomes*. Neonatal outcome variables were gestational age-specific SD scores for anthropometric measurements at birth: weight (grams), length (centimeters), and head circumference (HC) (centimeters). Neonates were weighed at birth by the midwife attending the childbirth, whereas birth length and HC were measured within the first 12 hr of life by a nurse in the hospital ward. Gestational age was established following the same procedure defined for ultrasounds. SD scores were calculated according to a customized random effects model taking into account maternal variables (preconception weight, height, and parity), paternal variables (height), and newborn variables (sex and gestational age at birth) (Mamelle et al. 2001). Detailed information about fetal growth modeling may be found in Supplemental Material, “Calculation of SD scores for neonatal parameters” and Figure S6.

*Assessment of air pollution exposure*. During pregnancy in each cohort, ambient levels of NO_2_ were measured with passive samplers (Radiello®; Radiello, Fundazione Salvatore Maugeri, Padua, Italy) installed in several sampling campaigns, each lasting 7 days and distributed over each study area in accordance with geographic criteria, taking into account the expected pollution gradients and the expected number of births.

The methodology applied for exposure modeling has been described previously (Estarlich et al. 2011; Iñiguez et al. 2009). Briefly, area-specific land-use regression (LUR) models of nitrogen dioxide (NO_2_) were developed to estimate residence-based exposures during pregnancy, using the average of the levels of NO_2_ registered across campaigns to represent an annual mean level, together with land use (agricultural, industrial, or urban), traffic-related variables, and altitude. Residential NO_2_ estimations from LUR were then adjusted to time of pregnancy for each woman, using daily records from the monitoring network stations covering the study area. Following this procedure, exposure to NO_2_ was estimated for the periods 0–12, 12–20, 20–34, 34–delivery weeks of gestation, and for the entire pregnancy.

*Covariates*. Detailed information on covariates was obtained from two questionnaires administered at 12 and 32 weeks of pregnancy: gestational weight gain (GWG) (in three categories: low/medium/high) following the Institute of Medicine (IOM) guidelines (IOM/NCR 2009); socio-occupational status [in three occupational categories according to most recent occupation (Domingo-Salvany et al. 2000)]; education (up to primary, secondary, and university); employment (yes/no); rural zone of residence (yes/no); country of origin (Spain/other); mother living with the father (yes/no); season of conception; alcohol consumption (yes/no); caffeine consumption (0 ≤ 100, > 100 < 200, ≥ 200 mg/day); vegetable, fruit, and energy intake (estimated from a food questionnaire in grams/day); active smoking during pregnancy (yes/no); type of cooking (electric/gas); heating (electric/gas); and use of a fume extractor in the kitchen (yes/no).

Environmental tobacco smoke exposure was assessed as passive exposure either at home, at work, or during leisure time and active smoking was considered as dichotomous (yes/no). Circulating 25-hydroxyvitamin D3 (vitamin D) was measured in maternal plasma at the first trimester by high-performance liquid chromatography.

*Statistical analysis*. Multivariate linear regression models were built to assess the relation between NO_2_ exposure and each outcome variable. First, a core model was built for each SD score using those covariates that were significant at a level of *p* < 0.2 in crude analyses as possible predictors. Following a forward procedure, all the covariates associated with outcomes at a level of *p* < 0.1 were introduced into the model (adjusted by cohort) except rural zone, which was a mandatory variable. Each exposure variable was then incorporated, and covariates changing the magnitude of the main effect by > 10% were also included. Variable selection was performed with the data set restricted to complete cases, but missing values within final models were not imputed. The percentage of such values was 4% (103 cases) on average, ranging from 0% to 11%. Models were examined for normality of regression residuals, collinearity (generalized variance-inflation factor > 2), extreme outliers (studentized residuals ≥ 4), and highly influential observations (Cook’s distance > 0.5).

Final models were applied to each cohort separately to account for the possible heterogeneity of the association between exposure and response variables, and the resulting estimates were combined by means of meta-analyses. Heterogeneity was quantified with the I-squared statistic (*I*^2^) (Higgins et al. 2003) and, if detected (*I*^2^ > 50%), the “random effect model” was used.

Generalized additive models, with penalized splines as smoothers, were used to explore the shape of the relation between fetal growth and NO_2_ exposure. Linearity was evaluated on the basis of the Akaike Information Criterion. The shape of the relationship between air pollution and size was nonlinear for EFW at all of the endpoints assessed, perhaps mediated by non-linear shapes in specific parameters [specifically, BPD at week 12, FL at week 20, and AC at week 34 (see Supplemental Material, Figures S7–S9)]. In consequence, average exposures to NO_2_ in each period were studied linearly (obtaining effect estimates by a 10-μg/m^3^ increase in exposure) and also dichotomized at the 66th percentile of NO_2_ exposure throughout the whole pregnancy (34.5 μg/m^3^).

Four sensitivity analyses were performed by re-running the cohort-specific models *a*) on the sample of term babies (around 95% of the initial sample); *b*) on the sample of mothers with coincident LMP-based and CRL-based gestational ages (around 80%); *c*) on the sample of women who spent ≥ 15 hr/day at home (around 60%); and *d*) by running a common model adjusted by GWG, season at conception, smoking, alcohol consumption, type of cooking, education, occupational status, and rural zone, which were the variables most frequently included (> 20%) in the set of models fitted by outcome and exposure.

Infant’s sex, type of cooking, season at conception, rural zone, GWG, alcohol and tobacco use, and fruit and vegetable intake (categorized at the median) were evaluated as potential effect modifiers. Effect modification was assessed through interaction terms and stratified analyses were performed.

The association was measured as the percentage of change in SD scores so as to enable comparison between outcomes. Statistical analyses were performed with R 3.1.3 (R Core Team 2014). Associations with a *p*-value < 0.05 are referred to as statistically significant.

## Results

*Subject and exposure characteristics*. Most of the 2,478 participating mothers (93.4%) had at least three examinations, providing a total of 7,602 ultrasounds. With these data, fetal growth curves were obtained for each parameter and cohort (see Supplemental Material, Figures S2–S5). Briefly, an association with sex was found for all fetal parameters except FL. BPD and FL showed a slight decline in growth toward the end of pregnancy, whereas AC was almost linear until term. As expected, the curve for EFW showed a fast increase in growth from mid-pregnancy onwards.

Exposure assignment was possible for 2,415 (97.5%) mothers, and estimated NO_2_ levels varied considerably among cohorts. Exposure and outcomes by cohort are described in Table 1.

**Table 1:** Ultrasound and NO_2_ exposure information: INMA Study, 2003–2008 (Spain).

Characteristic	Asturias	Gipuzkoa	Sabadell	Valencia	Overall
No. of mothers	478	603	611	786	2,478
No. of ultrasound examinations^*a*^
First trimester	461	600	602	775	2,438
Second trimester	494	592	609	811	2,506
Third trimester	606	586	622	844	2,658
Availability of CRL (%)	98.7	99.5	100.0	98.9	99.3
CRL-based GA (%)	11.3	10.3	12.9	12.3	11.8
GA at US:
First trimester	12.6 (11.3, 15.7)	12.4 (11.4, 13.6)	12.1 (10.9, 14.0)	12.4 (11.4, 13.4)	12.4 (11.3, 13.7)
Second trimester	20.7 (19.7, 21.9)	21.1 (19.8, 22.1)	21.1 (20.0, 22.4)	20.3 (19.1, 21.9)	20.7 (19.6, 22.1)
Third trimester	33.9 (31.0, 37.0)	34.1 (31.6, 35.3)	34.0 (32.3, 35.7)	32.3 (30.7, 38.1)	33.7 (31.0, 36.6)
No. of ultrasounds per mother (%)
2	9.8	6.1	3.3	7.6	6.6
3	61.5	92.9	93.9	77.2	82.1
≥ 4	28.7	1.0	2.8	15.1	11.3
Gestational age at birth (weeks)	39.6 (36.7, 41.7)	40.0 (37.4, 41.9)	39.9 (37.3, 41.7)	39.9 (36.6, 41.7)	39.9 (37.0, 41.7)*
Preterm deliveries (%)^*b*^	5.9	3.5	3.3	6.0	4.7*
Low birth weight (%)^*c*^	5.4	4.5	4.8	5.7	5.1
Birth weight (g)	3267.2 ± 474.8	3297.6 ± 456.5	3241.5 ± 436.6	3226.9 ± 527.3	3255.3 ± 479.6
Birth length (cm)	49.7 ± 2.1	49.0 ± 1.9	49.4 ± 2.0	50.1 ± 2.5	49.6 ± 2.2*
Birth HC (cm)	34.3 ± 1.4	34.7 ± 1.4	34.2 ± 1.3	34.0 ± 1.7	34.3 ± 1.5*
Mothers with exposure assignment (*n*)	475	592	564	784	2,415
NO_2_ levels^*d*^	23.1 ± 7.5	18.0 ± 6.0	35.7 ± 9.7	38.2 ± 11.7	29.7 ± 12.6*
Pearson correlation with NO_2_^*d*^
NO_2_ in weeks 0 to12	0.94	0.79	0.81	0.65	0.84
NO_2_ in weeks 12 to 20	0.93	0.88	0.79	0.82	0.87
NO_2_ in weeks 20 to 34	0.95	0.87	0.88	0.78	0.89
NO_2_ in weeks 34 to delivery	0.87	0.66	0.65	0.50	0.74
Percentages are presented for categorical variables. Mean ± SD or median (95% CI) are presented for continuous variables. ^***a***^In general, ultrasound examinations were complete, relating BPD, AC, and FL, except the ultrasound at week 12 in Asturias: *n *= 458 BPD data, *n *= 69 FL data, and *n *= 39 AC data. ^***b***^Preterm delivery: < 37 weeks of gestation. ^***c***^Low birth weight, < 2,500 g. ^***d***^NO_2_ for entire pregnancy. *Statistically significant differences among cohorts (*p* < 0.05).					

Cohort-adjusted analyses showed that more exposed mothers lived in urban areas, more often were non-Spanish, and more frequently used gas cooking and electric heaters and became pregnant in summer. Characteristics of mothers by NO_2_ levels are presented in the Supplemental Material, Table S1.

**Figure 1:**
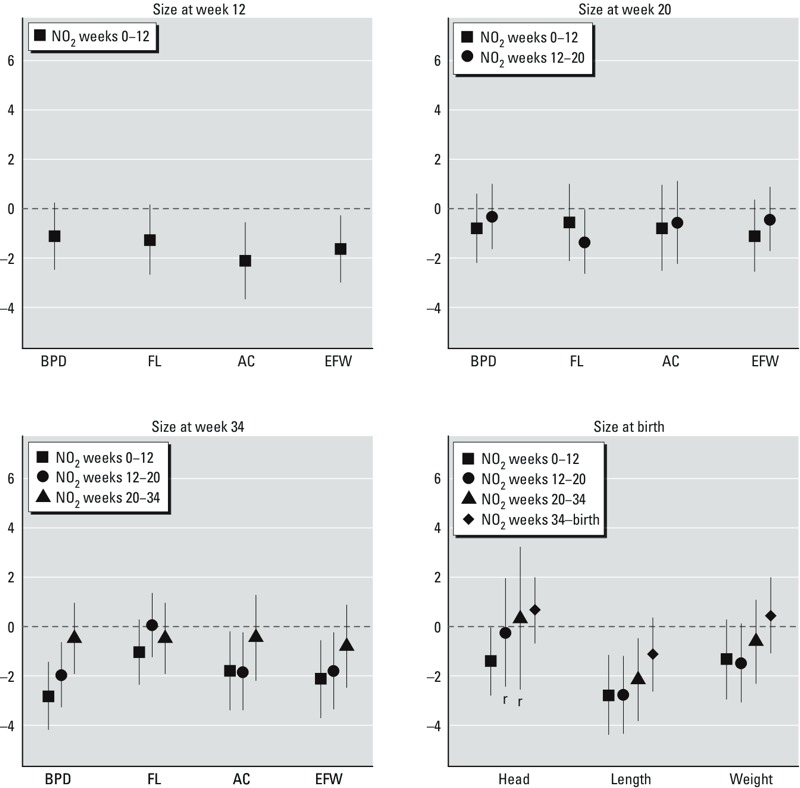
Maternal NO_2_ exposure (increase of 10 μg/m^3^) and fetal size in different stages of pregnancy: INMA study, 2003–2008. Percentage of difference in fetal (unconditional SD scores) and neonatal SD scores of size and their respective 95% CIs by a 10-μg/m^3^ increase in average exposure to NO_2_ during different windows of exposure at different stages of pregnancy. Estimates were obtained by meta-analyses under fixed or random effects models of cohort-specific estimates. The r identifies those meta-analyses performed under the random-effects model. Numeric estimates are presented in Supplemental Material, Table S2.

*Maternal NO_2_ exposure and scores of fetal growth*. An increase of 10 μg/m^3^ in NO_2_ levels during weeks 0–12 was inversely associated with AC and EFW growth at weeks 0–12 and with a nonsignificant decrease in FL growth at weeks 0–12 (Table 2). The same increase during weeks 0–12 and, to a lesser extent, during weeks 12–20 was associated with BPD, AC, and EFW at weeks 20–34, whereas exposure during weeks 12–20 was associated with a nonsignificant decrease in FL at weeks 12–20. Consistent with these negative associations, NO_2_ during early pregnancy was associated with significantly decreased size at week 34 in BPD, EFW, and AC, and a nonsignificant decrease in FL (Figure 1; see also Supplemental Material, Table S2).

**Table 2:** Exposure to NO_2_ in different stages of pregnancy and SD scores of fetal growth: INMA study, 2003–2008 (Spain).

Fetal score (weeks)	*n*	NO_2_ (per 10-μg/m^3^ increase)			NO_2_ > 34.5 μg/m^3^ (66th percentile)		
		% diff^*a*^ (95% CI)	*p*-Value^*b*^	*I*^2^(%)^*c*^	% diff^*a*^ (95% CI)	*p*-Value^*b*^	*I*^2^(%)^*c*^
BPD growth at 0–12
NO_2_ 0–12	2,389	–1.1 (–2.5, 0.2)	0.10	0	0.8 (–3.3, 4.9)	0.71	0
BPD growth at 12–20
NO_2_ 0–12	2,312	–0.2 (–1.5, 1.2)	0.84	0	0.0 (–4.2, 4.1)	0.98	23.6
NO_2_ 12–20	2,330	0.7 (–0.6, 2.0)	0.28	17.2	0.3 (–3.7, 4.4)	0.87	25.6
BPD growth at 20–34
NO_2_ 0–12	2,328	–2.6 (–3.9, –1.2)	< 0.01	0	–7.2 (–11.2, –3.1)	< 0.01	0
NO_2_ 12–20	2,325	–1.9 (–3.2, –0.6)	< 0.01	0	–6.5 (–10.5, –2.5)	< 0.01	0
NO_2_ 20–34	2,222	0.0 (–1.4, 1.5)	0.96	0	2.1 (–2.2, 6.3)	0.34	32.6
FL growth at 0–12
NO_2_ 0–12	2,310	–1.3 (–2.7, 0.2)	0.08	0	–0.2 (–4.5, 4.1)	0.92	0
FL growth at 12–20
NO_2_ 0–12	2,405	–0.4 (–2.0, 1.2)	0.48	0	–0.6 (–5.1, 3.8)	0.79	0
NO_2_ 12–20	2,406	–1.2 (–2.5, 0.1)	0.07	8.2	–4.5 (–8.4, –0.5)	0.03	0
FL growth at 20–34
NO_2_ 0–12	2,270	–0.8 (–2.1, 0.6)	0.26	0	–3.5 (–7.6, 0.7)	0.10	0
NO_2_ 12–20	2,252	0.1 (–1.5, 1.7)	0.92	0	–1.6 (–6.1, 2.9)	0.48	0
NO_2_ 20–34	2,266	–0.6 (–2.3, 1.1)	0.47	0	0.5 (–4.0, 5.0)	0.83	0
AC growth at 0–12
NO_2_ 0–12	2,402	–2.1 (–3.7, –0.6)	0.01	20.4	0.0 (–8.8, 8.8)	1.00	66
AC growth at 12–20
NO_2_ 0–12	2,250	0.8 (–0.9, 2.4)	0.37	14.1	0.7 (–3.7, 5.2)	0.76	0
NO_2_ 12–20	2,323	0.8 (–0.8, 2.4)	0.31	0	1.6 (–6.4, 9.4)	0.70	0
AC growth at 20–34
NO_2_ 0–12	2,323	–1.8 (–3.3, –0.2)	0.03	0	–4.3 (–8.8, 0.2)	0.06	5.9
NO_2_ 12–20	2,324	–1.7 (–3.3, –0.1)	0.04	0	–4.5 (–8.9, –0.1)	0.05	46.2
NO_2_ 20–34	2,141	–0.2 (–2.0, 1.6)	0.81	6.4	–1.0 (–8.8, 6.8)	0.81	52.1
EFW growth at 0–12
NO_2_ 0–12	2,399	–1.6 (–3.0, –0.3)	0.02	0	–1.2 (–5.3, 3.0)	0.58	31.8
EFW growth at 12–20
NO_2_ 0–12	2,243	0.7 (–0.9, 2.3)	0.42	0	2.6 (–1.9, 7.1)	0.26	0
NO_2_ 12–20	2,243	0.0 (–1.6, 1.6)	0.97	0	–2.7 (–7.2, 1.8)	0.24	0
EFW growth at 20–34
NO_2_ 0–12	2,309	–2.1 (–3.7, –0.5)	0.01	0	–6.3 (–10.7, –1.8)	0.01	0
NO_2_ 12–20	2,309	–1.5 (–3.1, 0.0)	0.06	0	–4.7 (–9.1, –0.3)	0.04	19.1
NO_2_ 20–34	2,252	–2.1 (–3.7, –0.6)	0.01	0	0.1 (–7.7, 7.8)	0.99	55.5
Cohort-specific models for BPD were adjusted for rurality, alcohol consumption, energy intake, employment, and weight gain. FL: rurality, vitamin D, energy intake, marital status, tobacco use, season, and GWG. AC: rurality, marital status, season, education, social class, employment, GWG, alcohol consumption, energy intake, and type of cooking. EFW: rurality, season, GWG, alcohol consumption, energy intake, employment, tobacco use, type of cooking, and education. ^***a***^Percent of difference in SD scores, obtained by combining cohort-specific estimates using meta-analysis. ^***b***^*p*-Value according to likelihood ratio (LR) test. ^***c***^*I*^2^ statistic of heterogeneity; estimates with *I*^2^ > 50% were derived using random effects models.							

**Figure 2:**
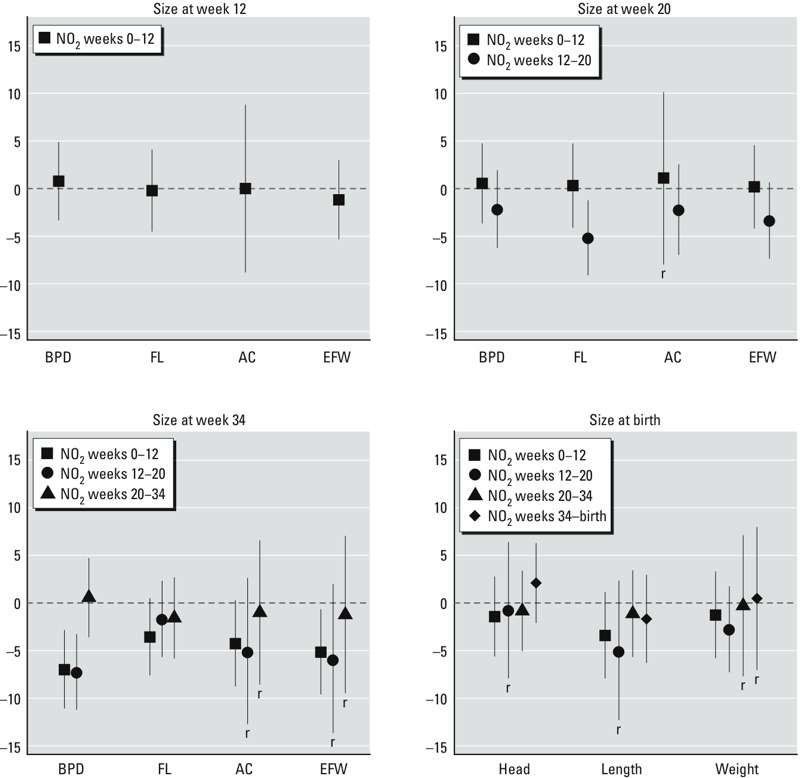
Maternal NO2 exposure (dichotomized at the 66th percentile) and fetal size in different stages of pregnancy: INMA study, 2003–2008. Percentage of difference in fetal (unconditional SD scores) and neonatal SD scores of size and their respective 95% CIs for NO2 over the 66th percentile (34.5 μg/m3) during different windows of exposure at different stages of pregnancy. Estimates were obtained by meta-analyses under fixed or random effects models of cohort-specific estimates. The r identifies those meta-analyses performed under the random-effects model. Numeric estimates are presented in Supplemental Material, Table S2.

Associations with average NO_2_ dichotomized at the 66th percentile (> 34.5 compared with ≤ 34.5 μg/m^3^) showed a similar pattern for growth at weeks 20–34 and size at week 34 (Table 2 and Figure 2). In contrast, high NO_2_ (> 34.5 μg/m^3^) during weeks 0–12 was not associated with AC and EFW growth at weeks 0–12, and high NO_2_ during weeks 12–20 was negatively associated with FL growth at weeks 12–20 and size of FL at week 20. Despite the indicator of exposure (linear or categorized), BPD showed the strongest negative associations, with estimated mean differences in size at week 34 of –2.8% [95% confidence interval (CI): –4.2, –1.4] and –7.3% (95% CI: –11.2, –3.3) in association with a 10-μg/m^3^ increase in NO_2_ and NO_2_ above versus below the 66th percentile, respectively.

Results remained stable when the sample was restricted to women with LMP-based gestational age. In general, the estimates after restricting the sample to women who spent at least 15 hr/day at home was slightly greater but less significant, and excluding preterm deliveries led to slightly clearer associations. Finally, the use of models with a consistent set of covariates also led to the same pattern except for slightly more significant associations on growth of FL and BPD at weeks 0–12 (see Supplemental Material, Table S3).

Stratified estimates of associations between NO_2_ during weeks 0–12 and size at week 34 for interactions with *p* < 0.1 are shown in Table 3. Associations of maternal exposure to NO_2_ during weeks 0–12 with fetal BPD and EFW at week 34 were stronger in active smokers than in nonsmokers. The negative association between NO_2_ and FL was stronger among women with a vegetable intake below versus above the median, whereas a positive association between NO_2_ and FL among women with high GWG during pregnancy was significantly different from the negative association among women with medium GWG. The negative association with EFW was weaker in high versus medium GWG women, and the association with AC was not modified (interaction *p* > 0.1) by any of the factors tested.

**Table 3:** Effect modification of NO_2_ impact on fetal growth: INMA study, 2003–2008 (Spain).

Parameter	Effect modifier	Category	*n*	Mean NO_2_	% diff^*a*^ (95% CI)	*p*-Value^*b*^	p_int_^*c*^
BPD	Active smoking	Overall	2,276	31.0	–2.9 (–4.3, –1.5)	< 0.01	
		No	1,545	30.5	–1.6 (–3.3, 0.1)	0.06
		Yes	731	32.0	–5.8 (–8.2, –3.5)	< 0.01	< 0.01
FL	Vegetable intake	Overall	2,340	30.8	–1.0 (–2.3, 0.3)	0.13
		≥ Median	1,170	31.1	–0.3 (–2.1, 1.6)	0.78
		< Median	1,170	30.4	–2.0 (–3.9, –0.8)	0.04	0.09
	GWG	Overall	2,340	30.8	–0.9 (–2.2, 0.4)	0.19
		Medium	863	30.1	–1.1 (–5.3, 3.1)	0.60
		Low	544	32.1	–1.1 (–3.7, 1.6)	0.43	0.81
		High	861	31.0	1.4 (–0.8, 3.6)	0.22	0.01
EFW	Active smoking	Overall	2,264	31.0	–2.2 (–3.8, –0.6)	0.01
		No	1,536	30.4	–1.5 (–3.5, 0.5)	0.14
		Yes	728	32.1	–3.9 (–6.6, –1.2)	< 0.01	0.03
	GWG	Overall	2,317	30.8	–2.1 (–3.7, –0.5)	0.01
		Medium	876	30.1	–2.7 (–5.3, –0.2)	0.04
		Low	563	32.0	–1.9 (–4.8, 0.9)	0.19	0.80
		High	878	30.8	–1.4 (–4.2, 1.4)	0.33	0.07
Overall estimates for NO_2_ at 0–12 weeks on size at week 34, restricted to valid cases for each potential effect modifier: sex, fruit and vegetable intake, type of cooking, alcohol consumption, tobacco use, rurality, weight gain, and season at conception. Effect estimates in each category are combined estimates by meta-analysis of cohort-specific estimates from the stratified analysis. ^***a***^Percentage of difference in SD scores. ^***b***^*p*-Value according to Wald test. ^***c***^Interaction *p*-value; results are shown if p_int_ < 0.1. The effect of NO_2_ on AC was not modified by anyone.							

*Maternal NO_2_ exposure and neonatal scores*. There were nonsignificant negative associations between a 10-μg/m^3^ increase in maternal exposure to NO_2_ during weeks 0–12 and HC at birth (–1.4%; 95% CI: –2.0, 0.0; corresponding to a mean difference of approximately 0.5 cm), and between NO_2_ during weeks 12–20 and birth weight (–1.5%; 95% CI: –3.1, 0.1; mean difference of approximately 50 g) (Table 4). Length at birth was significantly decreased in association with NO_2_ exposures during all exposure windows except 34 weeks–delivery, with a 10-μg/m^3^ increase in average NO_2_ over the entire pregnancy associated with a decrease of –3.2% (95% CI: –5.1, –1.3), corresponding to a decrease of approximately 1.5 mm relative to the mean length at birth.

**Table 4:** Exposure to NO_2_ during different periods of pregnancy and SD scores of neonatal anthropometry: INMA study, 2003–2008 (Spain).

Neonatal score (weeks)	*n*	NO_2_ (per 10-μg/m^3^ increase)			NO_2_ > 34.5 μg/m^3^ (66th percentile)		
		% diff^*a*^ (95% CI)	*p*-Value^*b*^	*I*^2^ (%)^*c*^	% diff^*a*^ (95% CI)	*p*-Value^*b*^	*I*^2 ^(%)^*c*^
HC
NO_2_ 0–12	2,284	–1.4 (–2.8, 0.0)	0.05	3.8	–1.4 (–5.6, 2.8)	0.50	0
NO_2_ 12–20	2,235	–0.2 (–2.4, 2.0)	0.84	50.6	–0.8 (–7.9, 6.4)	0.83	55.6
NO_2_ 20–34	2,138	0.3 (–2.6, 3.2)	0.82	65.8	–0.8 (–5.1, 3.4)	0.70	46.8
NO_2_ 34–delivery	2,191	0.7 (–0.7, 2.0)	0.32	12.5	2.1 (–2.1, 6.3)	0.33	0
NO_2_ pregnancy	2,233	–0.4 (–3.4, 2.7)	0.81	57.6	–3.5 (–7.7, 0.9)	0.12	17.4
Length
NO_2_ 0–12	2,286	–2.8 (–4.4, –1.2)	< 0.01	0	–3.4 (–7.9, 1.1)	0.14	0
NO_2_ 12–20	2,286	–2.8 (–4.3, –1.2)	< 0.01	0	–5.1 (–12.3, 2.3)	0.18	52.9
NO_2_ 20–34	2,284	–2.2 (–3.8, –0.5)	0.01	0	–1.1 (–5.7, 3.4)	0.63	0
NO_2_ 34–delivery	2,283	–1.1 (–2.6, 0.4)	0.14	0	–1.7 (–6.3, 2.9)	0.48	0
NO_2_ pregnancy	2,284	–3.2 (–5.1, –1.3)	< 0.01	0	–6.2 (–10.4, –1.8)	0.01	0
Weight
NO_2_ 0–12	2,317	–1.3 (–2.9, 0.3)	0.11	49.4	–1.3 (–5.8, 3.3)	0.58	0
NO_2_ 12–20	2,317	–1.5 (–3.1, 0.1)	0.07	0	–2.8 (–7.3, 1.7)	0.23	34.1
NO_2_ 20–34	2,261	–0.6 (–2.3, 1.1)	0.48	48.1	–0.3 (–7.7, 7.1)	0.94	51.8
NO_2_ 34–delivery	2,259	0.4 (–1.1, 2.0)	0.56	29.9	0.5 (–7.0, 8.0)	0.90	50.5
NO_2_ pregnancy	2,260	–1.2 (–3.1, 0.8)	0.24	35.6	–3.3 (–7.6, 1.1)	0.15	9.2
^***a***^Percent of difference in SD scores of neonatal anthropometry and its 95% CI, obtained by combining cohort-specific estimates using meta-analysis. ^***b***^*p*-Value according to likelihood ratio (LR) test. ^***c***^*I*^2^ statistic of heterogeneity; estimates with *I*^2^ > 50% were derived using random effects models.							

## Discussion

We estimated negative associations between maternal exposure to residential NO_2_ during pregnancy and growth of AC, EFW, and FL as early as week 12 of pregnancy. BPD after week 20 was strongly associated with exposure during weeks 0–12. The strongest and most consistent associations were related to exposure in early pregnancy, mainly during weeks 0–12. BPD was strongly affected, but from mid-pregnancy onward. Size at week 20 seemed to be associated with high levels of exposure (above vs. below the 66th percentile) during weeks 12–20, whereas size at week 34 was clearly associated with exposure during weeks 0–12 regardless of the type of indicator (linear or categorized).

As mentioned earlier, only six studies have estimated the impact of traffic-related air pollution on fetal biometry, and our results match with all but one (Hansen et al. 2008) on the stronger association between NO_2_ and head dimensions, measured as HC or BPD. The timing at which associations are stronger also coincides with those studies having ultrasound information in each trimester (Aguilera et al. 2010; Iñiguez et al. 2012; Slama et al. 2009; van den Hooven et al. 2012b). Relating to AC and EFW growth in early pregnancy, the study previously performed in the cohort of Valencia (Iñiguez et al. 2012), the Australian study (Hansen et al. 2008), and the Dutch study (van den Hooven et al. 2012b) also found an inverse association with FL in the second trimester of pregnancy, but associations with AC and EFW growth at week 12 have not been reported before. Associations with size at week 34 in all parameters were supported by our local INMA studies (Aguilera et al. 2010; Iñiguez et al. 2012). The Dutch study (van den Hooven et al. 2012b) found reduced FL and EFW at the third trimester (AC not examined).

Associations of neonatal outcomes with NO_2_ exposures were weaker than associations with ultrasound measures, except for birth length. In this respect, comparisons ought to be made with caution, because the characteristics measured are not exactly the same. Regarding length and weight, our results are consistent with the previously reported findings based on the same cohort (Estarlich et al. 2011). In this previous study, birth length and HC were examined directly, instead of gestational age and constitution-adjusted SD scores.

In relation to the timing of exposure to air pollution, our results suggest early pregnancy as the most harmful exposure window, and this joint INMA study, by increasing statistical power, provides support to the hypothesis that effects might be manifested immediately. Adverse effects on AC and EFW as early as week 12 of gestation are, to some extent, in contrast with the stated premise that head and bones of a fetus are more vulnerable during the first stages of pregnancy, whereas body mass accumulation could be more affected in late pregnancy. A possible explanation for these early effects on all parameters apart from BPD might result from a physiological adaptive response to hypoxia caused by toxic insults, in which brain development is preserved at the expense of a higher detriment of the other body segments. This pattern known as “brain sparing” has been described in association with maternal smoking during pregnancy and may lead to severe, even permanent, deficits in future health (Swanson et al. 2009).

One proposed biological mechanism by which air pollution may affect fetal growth is by causing a decrease in transplacental oxygen and nutrient transport (Kannan et al. 2006; Slama et al. 2008a). Poor placental vascularity is caused partially by dysregulation of gene expression in key angiogenic factors in early pregnancy, and this perturbed DNA transcription might in turn be related to air pollution exposure (Hansen et al. 2008). Placental development is particularly sensitive to pathology, and if it is disrupted, current and later placental function can be impaired. A recent study (Griffin et al. 2012) reported changes in umbilical blood flow in the third trimester after infections occurring before 20 weeks of gestation. This indicates that the effects might be observed with considerable delay in response to early exposure, which is in line with the pattern of our results.

Concerning the specificity of the relationship, our results suggest that adverse effects of air pollution on BPD and EFW were strengthened under active smoking. Synergy between air pollution and smoking might occur through different paths: by increasing vulnerability in the co-exposed (Mauderly and Samet 2009) or by acting on the same biological mechanisms. In this sense, it is well known that smoke constituents such as nicotine and carbon monoxide are, like NO_2_, strongly linked with fetal hypoxia (Haustein 1999). This synergic effect reinforces the need to promote healthy habits in mothers during pregnancy, with special emphasis on smoking cessation.

Regarding possible long-term consequences, it has been stated that restricted growth from mid- to late pregnancy predicts a higher risk of delayed infant development independently of postnatal growth (Henrichs et al. 2010). In turn, infants with reduced growth and adiposity in early childhood may have a higher tendency to experience later catch-up growth, strongly related to metabolic disorders such as obesity and insulin resistance (Crume et al. 2014). On the other hand, recent studies suggest that delayed development in specific parameters may have specific consequences for future health. In particular, poor prenatal head growth may represent a risk for behavioral disorders (Henrichs et al. 2009) and poor cognitive function (Yanney and Marlow 2004) in childhood; in a recent study (Eriksson et al. 2014), the adiposity rebound that is inversely related to the risk of infant and adult obesity has been found mostly associated with small head size at birth.

Some methodological considerations should be noted with regard to our study. First, our exposure estimates relied on the environmental modeling of residential outdoor levels. Consequently, some misclassification of personal exposure should be taken into consideration because outdoor levels of specific pollutants do not always reflect indoor levels, and people do not remain immobile inside their homes (Slama et al. 2008a). In this respect, indoor, occupational or in-transit exposures were at least partially addressed by adjusting for the available concomitant variables, such as exposure to environmental tobacco smoke, working status, or type of cooking, and in any case the misclassification of the assessment of air pollution exposure tends to be nondifferential (Ritz and Wilhelm 2008). Second, we confirmed or corrected LMP-gestational age by using an early CRL measurement. This procedure could lead to an underestimation of the adverse effects of air pollution if they took place before the CRL measurement (Slama et al. 2008b). In this respect, we preferred this conservative approach because the use of self-reported LMP for gestational dating is prone to large random measurement error, with more severe effects on estimates than those attributable to smaller systematic deviations (Jukic et al. 2008; Olsen and Fei 2008). Finally, the schedule of available ultrasounds implies that our study is unable to investigate air pollution effects in late pregnancy—an interesting period to study because it is that of the greatest fetal development. In this sense, this joint study allowed week 34 to be taken as a reference for the ultrasound measurement date in the third trimester rather than week 32, used in our first local analyses, the respective outcome indicators being a little more representative of late pregnancy.

The main strengths of our study were the use of repeated measurements of fetal biometry, allowing the identification of specific patterns of restricted fetal growth by body segment; the careful assessment of fetal growth, taking into account the individual growth potential of each fetus (Mamelle et al. 2001); the accurate exposure assessment based on a dense grid of measurements; exposure variability by including low and medium-high contaminated areas, in contrast to the majority of previous studies, which have been conducted in cities where ambient air pollution levels are relatively high; and, last, the availability and quality of the information at the individual level, collected using standardized protocols.

In conclusion, our study supports an adverse impact of maternal exposure to NO_2_ in the air during pregnancy on fetal growth from early pregnancy onward, even at levels deemed safe according to the limits established by current air quality standards. Overall, although air pollution exposure may be considered potentially modifiable, personal decisions to minimize exposure are almost unfeasible for the active population, thus reinforcing the need to implement and sustain cleaner air policies.

## Supplemental Material

(6.4 MB) PDF
