# Supplementary material for: Prenatal Exposure to NO2 and Ultrasound Measures of Fetal Growth in the Spanish INMA Cohort
Source: Environ Health Perspect. 2015 Jun 26;124(2):235–42. doi: 10.1289/ehp.1409423 (PMC4749079; doi:10.1289/ehp.1409423)
Supplement: (6.4 MB) PDF [file ehp.1409423.s001.acco.pdf]

Environ Health Perspect
DOI: 10.1289/ehp.1409423

Note to Readers: *EHP* strives to ensure that all journal content is accessible to all readers. However, some figures and Supplemental Material published in *EHP* articles may not conform to 508 standards due to the complexity of the information being presented. If you need assistance accessing journal content, please contact ehp508@niehs.nih.gov. Our staff will work with you to assess and meet your accessibility needs within 3 working days.

Supplemental Material

Prenatal Exposure to NO_2 and Ultrasound Measures of Fetal Growth in the Spanish INMA Cohort

Carmen Iñiguez, Ana Esplugues, Jordi Sunyer, Mikel Basterrechea, Ana Fernández-Somoano, Olga Costa, Marisa Estarlich, Inmaculada Aguilera, Aitana Lertxundi, Adonina Tardón, Mònica Guxens, Mario Murcia, Maria-Jose Lopez-Espinosa, and Ferran Ballester, on behalf of the INMA Project

Table of Contents

Fetal growth curves and calculation of SD scores
- Rationale
- General model description
- Conditional and unconditional centiles
- **Figure S1.** Correlation structure was an exponential variogram in all cases and random effects were never incorporated.
- **Figure S2.** Fetal growth curves for estimated fetal weight (EFW) in the four INMA-cohorts, INMA Study 2003-2008 (Spain)
- **Figure S3.** Fetal growth curves for abdominal circumference (AC) in the four INMA-cohorts, INMA Study 2003-2008 (Spain)
- **Figure S4.** Fetal growth curves for biparietal diameter (BPD) in the four INMA-cohorts, INMA Study 2003-2008 (Spain)

Figure S5. Fetal growth curves for femur length (FL) in the four INMA-cohorts, INMA Study 2003-2008 (Spain). There were no different FL curves by sex, since sex did not enter in any of the models.

Calculation of SD scores for neonatal parameters

Figure S6. Summary of models of neonatal parameters, INMA Study 2003-2008 (Spain). GA: gestational age in days, Random effects were never incorporated, therefore, estimates were performed using the generalized least-squares approach.

Table S1. INMA Study characteristics by NO_2 levels. INMA Study 2003-2008 (Spain)

Table S2. Exposure to NO_2 in different stages of pregnancy and SD scores of fetal size. INMA Study 2003-2008 (Spain)

Table S3. Results of sensitivity analyses. INMA Study 2003-2008 (Spain)

Figure S7. Shape of the relationship between exposure to NO_2 in weeks 0-12 and size of fetal parameters at week 12. INMA Study 2003-2008 (Spain). Shape estimated from a generalized additive model using a penalized cubic spline as a smoother. According to AIC, linearity was rejected for BPD and EFW, and the same pattern could be appreciated in these two cases. The vertical line is plotted at the 66th percentile of the distribution of NO_2 levels.

Figure S8. Shape of the relationship between exposure in the critical window of NO_2 (weeks 0-12 or weeks 12-20) and size of fetal parameters at week 20. INMA Study 2003-2008 (Spain). According to AIC, linearity was rejected for FL and EFW, and the same pattern could be appreciated in these two cases.

Figure S9. Shape of the relationship between exposure in the critical window of NO_2 (weeks 0-12) and size of fetal parameters at week 34. INMA Study 2003-2008 (Spain). According to AIC, linearity was rejected for AC and EFW, and the same pattern could be appreciated in these two cases.

References

Fetal growth curves and calculation of SD scores

Rationale

The purpose of building fetal growth curves in the INMA-Project is to establish a relationship between the given fetal characteristics and gestational age in the INMA population, taking into account those non-pathological biological factors that may influence the growth potential of each fetus, and then to use these curves to estimate possible intrauterine restrictions of growth at several times within pregnancy.

Theoretically, considering the constitutional potential of each fetus should allow us to discriminate better between small fetuses (related to the size of the general population) and reduced growth (related with the characteristics of the fetus itself) (Mamelle et al. 2001).

General model description

The data for a single fetal parameter consist of vectors of observations:

$$\left\{ \left(Y_{ij}; T_{ij}; C_i^p; M_i^q \right), \quad i = 1,...,n; \; j = 1,...,N_i; \; p = 1,...,P; q = 1,...,Q \right\},$$

where T_{ij} is the jth time-point in days for the ith fetus and Y_{ij} is the corresponding measurement. $\left(C_i^1,...,C_i^P\right)$ are the paternal and fetal characteristics identified in the literature as possibly influencing fetal growth. $\left(M_i^1,...,M_i^Q\right)$ are dichotomous variables tagging pregnancies with at least two consecutive ultrasounds performed too close together in time under different definitions of "too close". The response variable Y_{ij} was transformed searching for the linearity of the within-subject relationship with time. The transformation of the response, suggested in Gurrin et al. (2001) and Royston and Altman (1994), is a modification of the power transformation suggested by Box and Cox which takes: $Y_{ij}^{(\lambda)} = \begin{cases} Y_{ij}^{\lambda} & \lambda \neq 0 \\ \log(Y_{ij}) & \lambda = 0 \end{cases}$.

For the same purpose, we tested a polynomial of entire order until 3 in T_j or a low-order fractional polynomial, described by Royston and Altman (1994) in order to model the shape of response over time.

The full model is thus written:

$$Y_{ij}^{(\lambda)} = X_{ij}\beta + Z_{ij}b_i + \varepsilon_{ij}$$

where:

- $X_{ij} = \lfloor 1,\ p(T_{ij}),\ C_i^1,\ldots,C_i^P,\ T_{ij}\times C_i^1,\ldots,T_{ij}\times C_i^P \rfloor$ and β is the corresponding vector of fixed coefficients to be estimated.

- $p(T_{ij})$ is a subset of the columns of $\lfloor T_{ij}, T_{ij}^2, T_{ij}^3 \rfloor$ or an element of the class of fractional polynomial of degree 2: $\lfloor T_{ij}^{p1}, T_{ij}^{p2} \rfloor$, with $p1, p2 \in \left\{-2,-1,-\frac{1}{2},0,\frac{1}{2},1,2,3\right\}$ and with pi=0 corresponding to the logarithmic transformation.

- $\left(C_i^1,\ldots,C_i^P\right)$ are the subset of the biological determinants considered: maternal and paternal height, maternal and paternal weight or body mass index (BMI), maternal age, parity, country of origin, and fetal sex. We checked whether they were reasonable under different metrics. $\left(T_{ij}\times C_i^1,\ldots,T_{ij}\times C_i^P\right)$ are their interactions with the time at measurement.

- $Z_{ij} = \lfloor 1, T_{ij} \rfloor$ represents the individual deviations from the mean of the fetal parameter for the population: constant deviations and linear change over gestation are allowed. b_i is the corresponding vector of random effects which is estimated for each fetus, and whose distribution across the fetal population is assumed to be bivariate normal: $b_i = (b_{0i},\ b_{1i}) \propto N(0, D)$. b_i is assumed to be independent among the subjects.

- ε_{ij} is the random variable representing the deviation in size at each time j on the ith fetus from the mean size. ε_i are called within-subject errors and are assumed to be bivariate normal: $\varepsilon_i = (\varepsilon_{i1},\ldots,\varepsilon_{iN}) \propto N(0,\sigma^2\Lambda_i)$. The specification of the model additionally requires the independence of within-subjects residuals between subjects.

Commonly, although not necessarily, the independence of ε_{ij} within subjects (that is, Λ_i=I) is also specified, but in our case we used the extended model to allow for: 1) heteroscedasticity, and 2) autocorrelation of within-subject errors. This was performed in the following way:

1) $\sigma^2\Lambda_i(j,j) = \mathrm{var}(\varepsilon_{ij}) = \sigma^2 \cdot g(T_{ij}, C_i, M_i, \delta)$,

where g is a function of at least one of the following variables: time, biological covariates, and

the dummies identifying subjects with an atypical sequence of ultrasound times:

$$M_i^q = \begin{cases} 0 & |T_{ij} - T_{ik}| > q, \ \forall j,k \\ 1 & \text{other case} \end{cases} \qquad q = 18, 21, 30 \text{ days}$$

As has been said before, M^q are factors tagging mothers with at least two ultrasounds that were performed too close together in time; these variables were checked to see wheter they caused heteroscedasticity following the idea that series with an unusual schedule within routine clinical practice could respond to a special situation or include some mistake, thus being less representative of the general population.

Several possibilities that can be used as g functions are implemented in R by default. In our models, one of the M_i^q was commonly selected as influencing variance, in which case the g function consists in simply assigning different variances for each category. In all cases, the greater assigned variance matched the category of atypical mothers.

2) $\sigma^2 \Lambda_i(j,k) = \text{cor}(\varepsilon_{ij}, \varepsilon_{ik}) = \sigma^2 f(d_{jk}, \phi)$, 2) $\sigma^2 \Lambda_i(j,k) = \text{cor}(\varepsilon_{ij}, \varepsilon_{ik}) = \sigma^2 f(d_{jk}, \phi)$,

where f is a function which usually decreases with the distance between observations: $d_{jk} = |T_{ij} - T_{ik}|$, ϕ parameter to be estimated. Different functions are available in R to be used here as f, including well known from time-series or spatial data theory, are: AR, MA, ARMA, CAR, or exponential or Gaussian variograms. In our models, the most commonly selected function was the exponential variogram representing an exponential decay in the correlation between observations with the difference in time between them, that is, $f(d_{jk},\phi)= 1-\exp(d_{jk}/\phi)$**.**

Conditional and unconditional centiles

The subsequent development and notation closely follow that of Royston (1995) and Gurrin et al. (2001) and further information may be found there. For each fetal dimension, once the corresponding linear mixed model was adjusted, the customized deviation of size in the ith fetus at time j, in relation to its potential, may be obtained in the usual way by employing the modeled mean and variance of the transformed response, $Z=Y^{(\lambda)}$, at time j:

$$z_{ij} = \frac{Z_{ij} - E[Z_{ij}]}{\text{Var}[Z_{ij}]}$$

These are unconditional relative deviations, describing only a deviation in size, as any other information except the time and the characteristics of the fetus itself has been considered.

The linear mixed model assumes that the series of measurements within a given fetus have a multivariate normal distribution, hence implying that both marginal and conditional distributions of each pair of measurements Z_2 and Z_1 are univariate normal and the conditional distribution of Z_2 given Z_1 is univariate normal with mean and variance: $\mu_{2|1} = E[Z_2 \mid Z_1] = \mu_2 + \frac{\sigma_{12}^2}{\sigma_1^2}(Z_1 - \mu_1)$,

$\sigma_{2|1}^2 = \mathrm{Var}[Z_2 \mid Z_1] = \sigma_2^2 - \frac{\sigma_{12}^2}{\sigma_1^2}$

The conditional deviation defined by:

$$z_{2|1} = \frac{Z_2 - \mu_{2|1}}{\sigma_{2|1}^2}$$

is the standardization of the transformed response at time $T_{2,}$ according to its conditional mean and variance at time T_2 given the observed value at time T_1.

That is, the status of the ith fetus at time T_1 is taken into consideration to update the mean and variance that should be used as a reference in T_2.

In our case, unconditional centiles were calculated for j=12, 20 and 34 weeks of gestation and conditional centiles were calculated for the intervals: 12–20, 12–34 and 20–34 weeks. Most women had ultrasound measurements at approximately 12, 20 and 34 weeks but not exactly at these points. Searching for the synchronization of outcomes, we calculated SD scores at a particular time, using the prediction at this particular time point conditioned to the nearest measure. That is, for example, if an ultrasound was performed at week 19, the SD score for week 20 was calculated in the standard way but using the prediction (from the modeled curve) of size at week 20 given the attained size at week 19 instead of the measured size at week 19. This procedure was used to prevent an increase in random error caused by the misalignment of measurements and by itself guarantees a complete data basis with SD close to 0 when there is a gap in the planned schedule of ultrasounds at weeks 12, 20 and 34.

In all models, after adjusting for covariates and the variance-covariance structure for within-subject errors, random effects were not necessary; so model estimation was performed by using

the generalized least-squares approach (see point 7) of steps of the modeling procedure, presented below). The best-fitting models always included a 2nd or 3rd degree polynomial of gestational age. Pre-pregnancy weight, age of mother, fetus sex and father's height were the most frequently included covariates (see Figure S1). Models always incorporated an exponential variogram of the days between ultrasound scans as correlation structure and heteroscedasticity was commonly found according to one of the factors M^q.

For each fetal dimension in each cohort dataset:

1.) Estimation of λ for Box-Tidwell transformation of response: Searching for the normality in residuals of groups by a cubic polynomial of T. Functions: *aov* and *boxcox* (MASS library) (Gurrin et al. 2001).

2.) Selection of the best function to describe the change of parameters over time, that is, the specification of p(T). Functions: *glm* and *mfp* (mfp library). Selection criterion: minimum AIC.

3.) Introduction of covariates at intercept: applied on all but M_i. Method: forward. Function: gls (nlme library), in close connection with GEE (Pinheiro and Bates 2000), ML estimation. Selection criterion: LR test (*p*-value<0.05).

4.) Introduction of covariates interacting with time: as in 3.) and re-evaluation of covariates at intercept.

5.) Specification of correlation structure for within-subject errors. Covariates considered: T. Possible structures: CAR and variograms: exponential, gaussian, spherical, linear, rational squared (Pinheiro and Bates 2000). Selection criteria: minimum AIC over those structures which were significant (LR test; *p*-value<0.05) and presented no over-fitting (pACF of normalized residuals inspection). Again, re-evaluation of terms currently in the model.

6.) Specification of variance structure for within-subject errors. Covariates considered: T, C, M. Possible structures: varPower (for continuous covariates), varIdent (for categorical covariates) or a combination (Pinheiro and Bates 2000). Selection criterion: minimum AIC over those structures which were significant (LR test; *p*-value<0.05). Again, re-evaluation of terms actually in the model.

7.) Random-effects incorporation: tested if only at intercept, only at slope or in both terms. Functions: *gls* (nlme library), *lme* (nlme library). Selection criteria: Conditional F-test comparing with the full gls model re-fitted by REML (p-value<0.05) and no over-fitting given by the previously included correlation structure.

8.) Diagnosis: Normalized residuals should be N(0,I), random effects should be N(0,D), and independent among subjects. If necessary, go back to 2.).

9.) Prediction of aligned estimates to be used as observations at weeks 12, 20 and 34, and to obtain SD scores as previously described.

BPD	Asturias	Gipuzkoa	Sabadell	Valencia
l	0.64	0.62	0.78	0.67
P(T) order	2	3	3	2
Mother age		x		x
Mother height	x	x	x	
Father height				
Mother weight/bmi		x		x
Father weight/BMI	x	x	x	x
Parity	x			x
Country of origen			x	x
Sex	x	x	x	x
Variance structure	M^{18}	country, parity		M^{21}

FL	Asturias	Gipuzkoa	Sabadell	Valencia
l	0.69	0.74	0.80	0.79
P(T) order	3	3	3	3
Mother age		x	x	x
Mother height	x	x	x	x
Father height	x	x		x
Mother weight/bmi	x			x
Father weight/BMI			x	
Parity				x
Country of origen		x	x	
Sex				
Variance structure	M^{30}, parity	M^{30}, T	M^{21}, T	T

AC	Asturias	Gipuzkoa	Sabadell	Valencia
l	0.34	0.45	0.59	0.44
P(T) order	3	3	3	3
Mother age		x	x	x
Mother height			x	
Father height	x			x
Mother weight/bmi	x	x	x	x
Father weight/BMI		x	x	
Parity				
Country of origen		x	x	
Sex	x	x	x	x
Variance structure	M^{30}, T		parity	M^{30}

EFW	Asturias	Gipuzkoa	Sabadell	Valencia
l	log	log	0.06	log
P(T) order	3	2	3	2
Mother age		x	x	x
Mother height		x	x	
Father height	x			x
Mother weight/bmi	x	x		x
Father weight/BMI			x	
Parity				
Country of origen		x	x	x
Sex	x	x	x	x
Variance structure	M^{21}, parity	parity, T	M^{30}	M^{30}

Supplemental Material, Figure S1. Summary of models of fetal parameters by cohort, INMA Study 2003-2008 (Spain). Correlation structure was an exponential variogram in all cases and random effects were never incorporated.

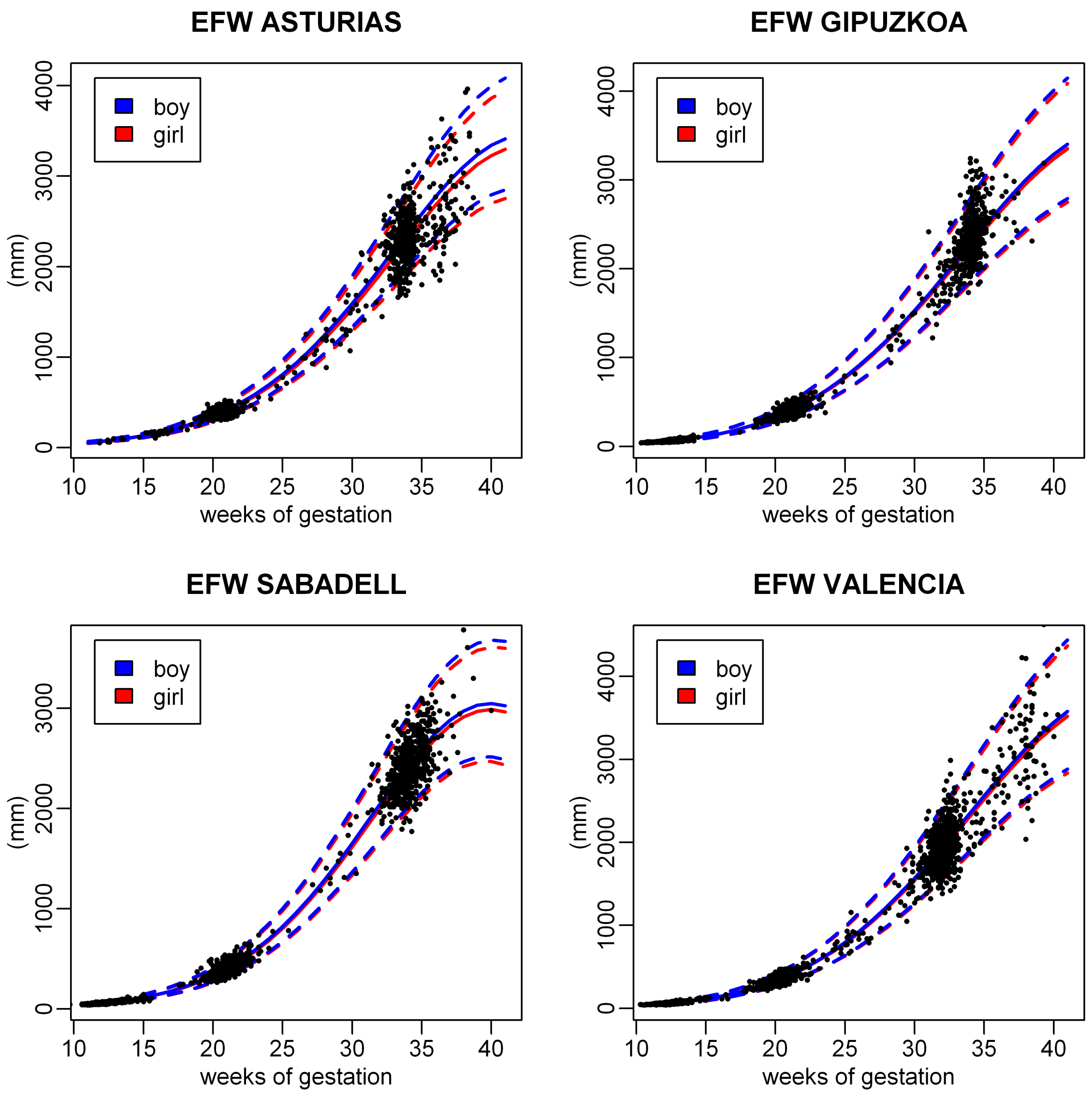

Supplemental Material, Figure S2. Fetal growth curves for estimated fetal weight (EFW) in the four INMA-cohorts, INMA Study 2003-2008 (Spain)

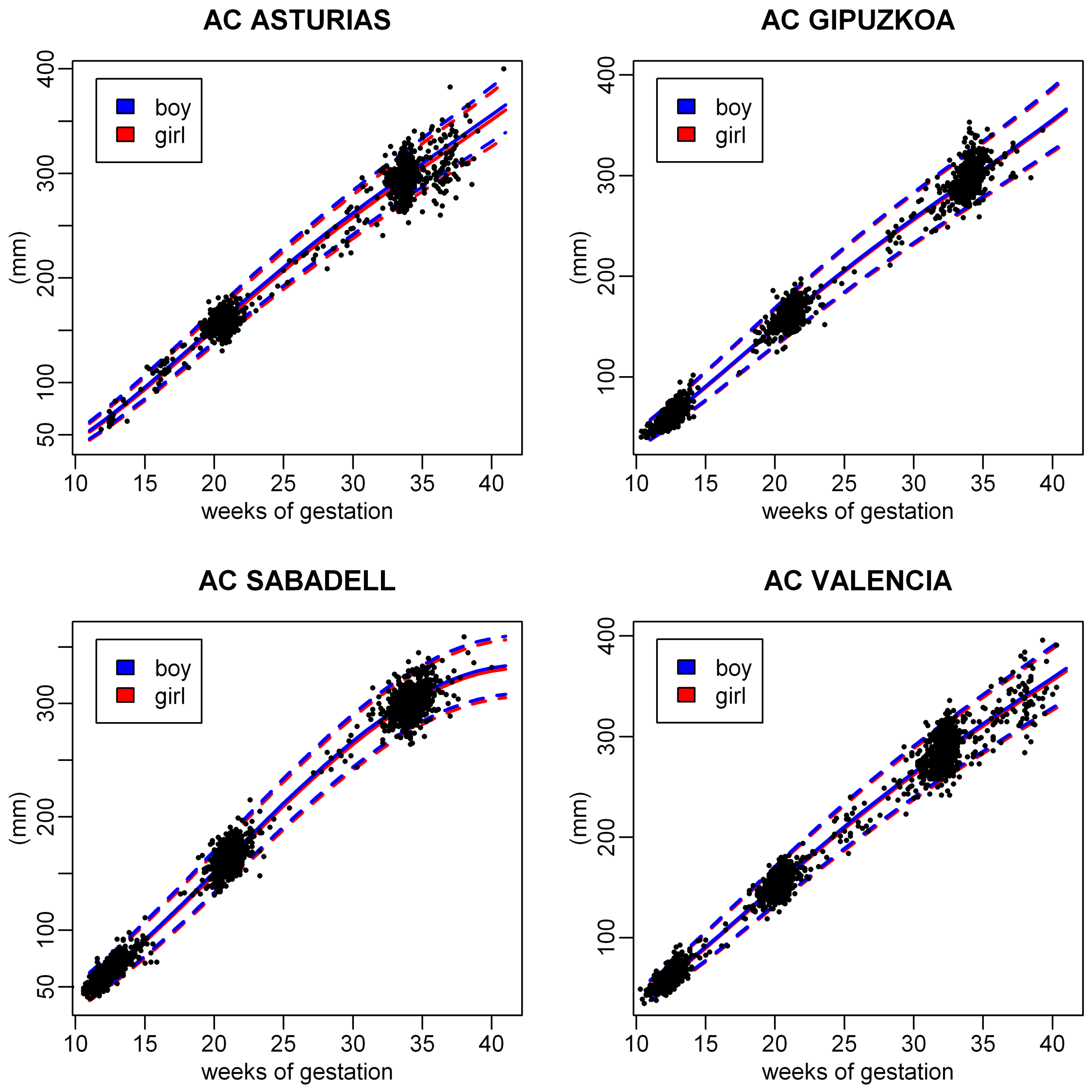

Supplemental Material, Figure S3. Fetal growth curves for abdominal circumference (AC) in the four INMA-cohorts, INMA Study 2003-2008 (Spain)

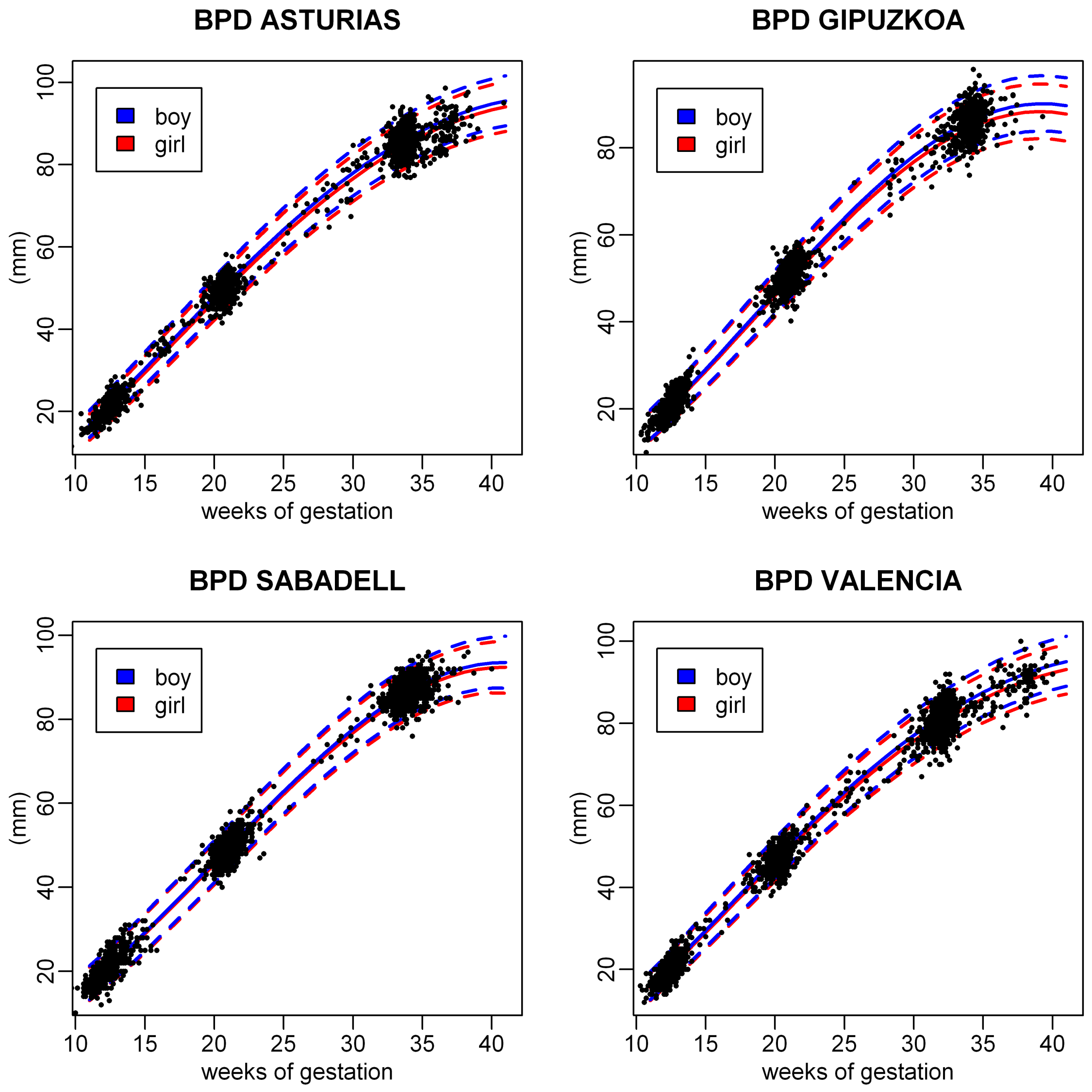

Supplemental Material, Figure S4. Fetal growth curves for biparietal diameter (BPD) in the four INMA-cohorts, INMA Study 2003-2008 (Spain)

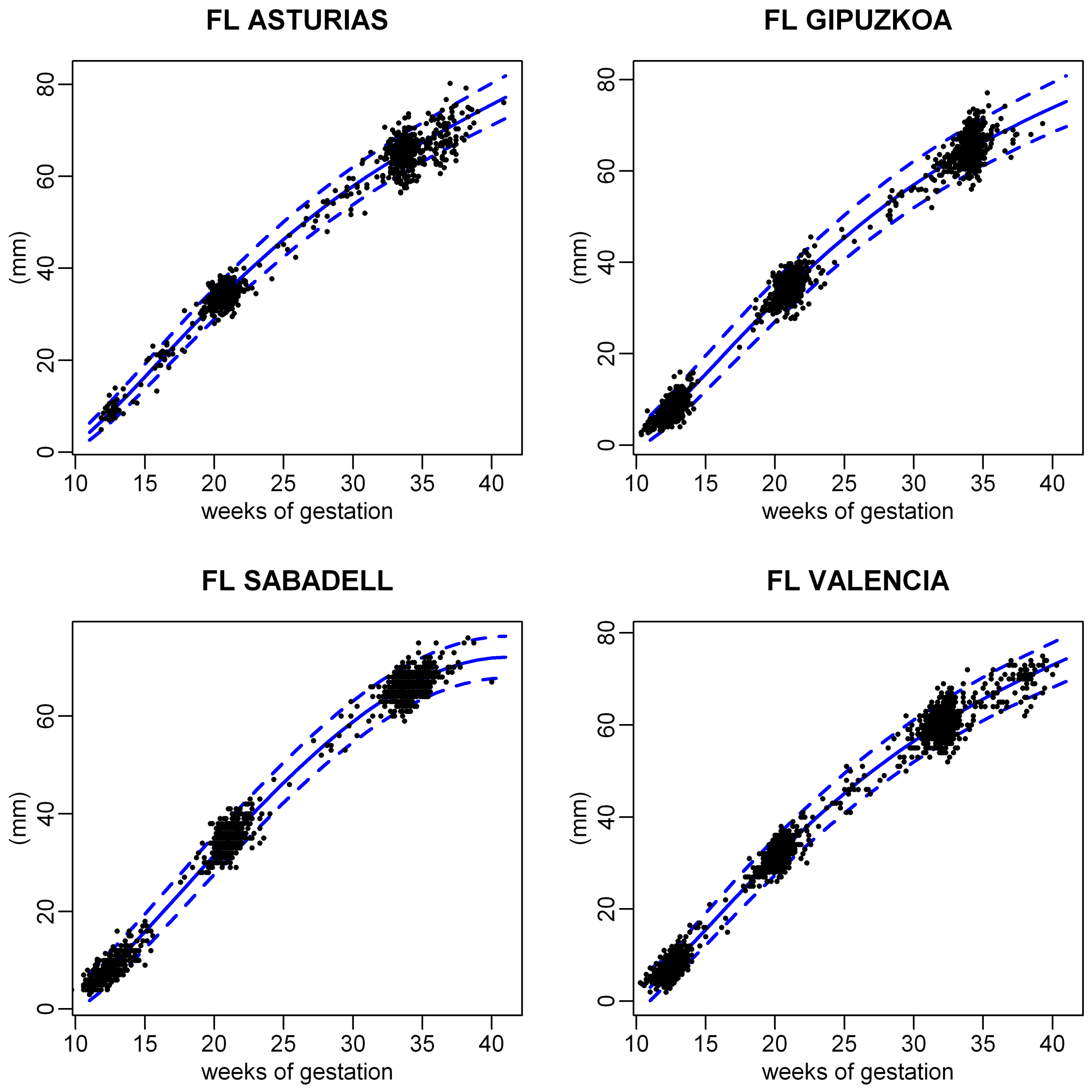

Supplemental Material, Figure S5. Fetal growth curves for femur length (FL) in the four INMA-cohorts, INMA Study 2003-2008 (Spain)

There were no different FL curves by sex, since sex did not enter in any of the models.

Calculation of SD scores for neonatal parameters

We fitted models for neonatal outcomes (weight, length and head circumference [HC] at birth) adjusted by potential biological determinants of fetal growth with the same aim as was previously described for fetal curves. That is, considering physiological determinants of fetal growth as explanatory variables would allow to us to obtain an individualized rather that a population-based standard for size at birth.

Briefly, we followed a similar procedure to that outlined for ultrasounds. A linear mixed model (Pinheiro and Bates 2000) was again used as theoretical model, but in this case the data for each parameter were analyzed jointly, thereby allowing for random effects by cohort on intercept and covariate effects.

The set of potential covariates considered was: gestational duration (days), cohort, maternal and paternal height, maternal and paternal weight or BMI, maternal age, parity, maternal country of origin, and fetal sex.

As in fetal models, the response variable was transformed using the power transformation suggested by Box and Cox. Second and 3rd entire order polynomials and fractional order polynomials of degree 2 (Royston and Altman 1994) were considered for the relationship with gestational age. Potential determinants of growth and their interactions with gestational age were tested to be included in the model (conditional LR test; $p<0.05$) and the basic model was extended to allow for heteroscedasticity explained by the covariates, in two ways: equal or different effects by cohort.

Finally, random effects by cohort on intercept and slope (covariates) in the model were tested by the LR test ($p<0.05$). The goodness of fit was assessed by considering the normality and independence of residuals. Extreme outliers were dropped (>4SD) and tests were conducted for the presence of influential data. As in fetal curves, after adjusting for covariates and modeling the variance structure, random effects were not necessary, although the categorical variable "cohort" was always incorporated. A summary of terms in the models for birth, length and HC at birth is presented in Figure S6.

SD scores were calculated using this model in the standard way as described in the previous section "Fetal growth curves and calculation of SD scores."

Models	Weight	Length	HC
1	0.64	0.62	0.78
P(GA) order	2	3	3
Cohort	x	x	x
Mother age			
Mother height	x	x	x
Father height	x	x	x
Mother weight/bmi	x	x	x
Father weight/BMI			
Parity	x	x	x
Country of origin	x	x	
Sex	x	x	x
Variance structure	Sex	Sex by cohort	GA by cohort

Supplemental Material, Figure S6. Summary of models of neonatal parameters, INMA Study 2003-2008 (Spain). GA: gestational age in days, Random effects were never incorporated, therefore, estimates were performed using the generalized least-squares approach.

Supplemental material, Table S1. INMA Study characteristics by NO_2 levels. INMA Study 2003-2008 (Spain)

Variable	N	(%)	NO_2 (μg/m^3) Mean (SD)	p[a]	NO_2>Q_{66} [c] (%)	p[b]	Variable	N	(%)	NO_2 (μg/m^3) Mean (SD)	p[a]	NO_2>Q_{66} [c] (%)	p[b]
**Maternal Age (years)**	2477			0.11		0.34	**Caffeine intake (mg/day)[d]**	2456			0.40		0.94
<25		7.2	35.3±13.0		51.2		<100		66	30.5±12.8		34.3	
25-29		32.1	30.0±12.7		33.9		100-200		22.5	28.6±12.0		30.3	
30-34		42.2	28.9±12.3		30.2		≥200		11.6	27.6±12.3		25.9	
35+		18.4	28.8±12.5		27.7		**Energy intake (g/day)[d]**	2478			0.25		0.49
**Maternal Height (cm)**	2477			0.74		0.98	<10.8		24.1	27.3±12.3		26.0	
<160		27.8	31.1±12.9		36.4		10.8-11.2		49.9	29.2±12.6		30.9	
160-165		33.3	29.7±12.5		31.8		11.2+		26	32.9±12.4		41.3	
165+		38.9	28.7±12.4		30.2		**Vegetable intake (g/day)[d]**	2478			0.93		0.89
**Pre-pregnancy weight (kg)**	2478			0.14		0.30	<7.2		25.1	30.1±12.4		33.4	
<55		25	30.7±12.8		35.8		7.2-8.1		50.6	29.0±12.8		31.3	
55-70		54.1	29.1±12.5		30.6		8.1+		24.3	30.7±12.5		34.0	
70+		20.9	30.1±12.8		33.4		**Fruit intake (g/day)[d]**	2478			0.05*		0.36
**GWG**	2391			0.56		0.85	<7.7		25.5	31.5±12.7		38.2	
Low		24.1	30.2±12.6		32.2		7.7-8.7		48.4	28.6±12.4		29.5	
Medium		38.1	29.4±12.8		31.6		8.7+		26.1	30.1±12.8		32.3	
High		37.8	30.2±12.5		34.8		**25-hydroxyvitamin D(3)**	2381			0.06		0.09
**Education level**	2473			0.52		0.95	<4.4		23	28.3±11.7		26.2	
Primary		24.5	32.8±12.5		42.0		4.4-5.2		50.7	30.1±12.8		34.2	
Secondary		41.3	29.9±12.5		33.1		5.2+		26.3	29.9±12.9		33.3	
University		34.2	27.3±12.3		24.7		**Cooking**	2389			<0.01*		<0.01*
**Social class[e]**	2477			0.82		0.69	Electric		55.5	25.5±11.5		18.7	
I+II		31.3	27.6±12.4		25.7		Gas		44.5	35.2±12.0		50.4	
III		25.2	30.5±12.7)		34.8		**Heater**	2365			<0.01*		<0.01*
IV+V		43.5	30.7±12.6)		36.0		Electric		31.1	35.6±12.6)		52.5	

**Living with the father**	2477			0.37		0.54
Yes		98.3	29.7±12.6)		32.2	
No		1.7	31.7±11.6)		46.3	
**Employed in pregnancy**	2478			0.25		0.61
Yes		83.6	29.7±12.8)		32.8	
No		16.4	29.9±11.9)		31.0	
**Country of origin Spain**	2470			0.04*		0.06
Yes		91.8	29.2±12.6)		30.9	
No		8.2	34.9±12.3)		51.0	
**Rural**	2470			<0.01*		<0.01*
Yes		5.8	16.9±6.2)		0.0	
No		94.2	30.5±12.5)		34.5	
**Active smoking**	2411			0.05*		0.48
Yes		31.7	30.9±13.0)		37.1	
No		68.3	29.3±12.4)		30.7	
**Passive smoking**	2399			0.64		0.79
Yes		62.5	30.8±12.9)		36.2	
No		37.5	28.1±12.1)		26.6	
**Alcohol consumption**[d]	2456			0.32		0.07
Yes		12.7	32.8±13.6)		45.2	
No		87.3	29.3±12.4)		30.6	
Gas		63.5	27.0±11.6)		22.9	
Other		5.4	28.5±12.5)		27.0	
**Extractor**	2307			0.02*		0.09
Yes		72.4	29.1±12.8)		30.5	
No		27.6	31.6±12.0)		37.7	
**Season at conception**	2478			<0.01*		<0.01*
Winter		25.4	29.4±11.6)		31.9	
Spring		25.6	29.2±12.3)		31.8	
Summer		25.7	30.3±13.5)		33.3	
Autumn		23.3	30.0±13.0)		32.9	
**Gender**	2474			0.85		0.20
Female		48.4	29.6±12.5)		31.2	
Male		51.6	29.8±12.8)		33.7	
**Primiparous**	2476			0.82		0.36
Yes		56.2	29.7±12.8)		32.7	
No		43.8	29.7±12.4)		32.1	
**Gestational age (weeks)**	2475			0.17		0.73
<37	2475	4.6	31.8±14.3)		35.1	
37-39		23.1	29.0±12.5)		30.0	
39+		72.2	29.8±12.5)		33.1	

Mean±SD or percentages are presented for all mothers included in the association analysis. (a) p-value of LR test, linear regression, models only adjusted by cohort, * p-value<0.05. (b) p-value of LR test, logistic regression, models adjusted by cohort,* p<0.05. (c) Q_{66}=34.5 µg/m^3, 66th percentile of NO_2 distribution. (d) Models also adjusted by energy intake. Fruit intake, vegetable intake and vitamin D were log2 transformed and classified in tertiles for description. (e) Class I: managerial jobs, senior technical staff, and commercial managers; Class II: skilled non-manual workers; and Class III: manual workers.

Supplemental material, Table S2. Exposure to NO_2 in different stages of pregnancy and SD scores of fetal size. INMA Study 2003-2008 (Spain).

Fetal score	N	NO_2 (per 10μg/m^3 increase)			NO_2> 34.5 μg/m^3 (66th percentile)		
		% diff[a] (95% CI)	p[b]	I^2(%)[c]	% diff[b] (95%	p[c]	I^2(%)[d]
**BPD size at 12**							
NO_2 0-12	2389	-1.1(-2.5, 0.2)	0.10	0	0.8(-3.3, 4.9)	0.71	0
**BPD size at 20**							
NO_2 0-12	2315	-0.8(-2.2, 0.6)	0.27	24.2	0.5(-3.7, 4.7)	0.80	10.5
NO_2 12-20	2315	-0.3(-1.6, 1.0)	0.64	24.8	-2.2(-6.2, 1.9)	0.30	47.1
**BPD size at 34**							
NO_2 0-12	2332	-2.8(-4.2, -1.4)	<0.01	0	-7.0(-11.1, -2.9)	<0.01	0
NO_2 12-20	2330	-2.0(-3.3, -0.6)	<0.01	0	-7.3(-11.2, -3.3)	<0.01	0
NO_2 20-34	2266	-0.5(-1.9, 1.0)	0.52	0	0.6(-3.6, 4.7)	0.79	20.8
**FL size at 12**							
NO_2 0-12	2310	-1.3(-2.7, 0.2)	0.08	0	-0.2(-4.5, 4.1)	0.92	0
**FL size at 20**							
NO_2 0-12	2405	-0.6(-2.1, 1.0)	0.48	0	0.3(-4.1, 4.7)	0.89	0
NO_2 12-20	2406	-1.4(-2.6, 0.0)	0.04	0	-5.0(-8.9, -1.0)	0.01	0
**FL size at 34**							
NO_2 0-12	2340	-1.0(-2.4, 0.3)	0.13	0	-3.6(-7.6, 0.5)	0.08	0
NO_2 12-20	2267	0.1((-1.2, 1.4)	0.92	0	-1.7(-5.7, 2.3)	0.41	0
NO_2 20-34	2336	-0.5(-1.9, 1.0)	0.50	0	-1.6(-5.8, 2.7)	0.47	3.5
**AC size at 12**							
NO_2 0-12	2402	-2.1(-3.7, -0.6)	0.01	20.4	0.0(-8.8, 8.8)	1.00	66
**AC size at 20**							
NO_2 0-12	2130	-0.8(-2.5, 1.0)	0.37	0	1.1(-8.8, 10.1)	0.81	63.9
NO_2 12-20	2131	-0.6(-2.2, 1.1)	0.52	0	-2.2(-6.9, 2.5)	0.36	35.4
**AC size at 34**							
NO_2 0-12	2324	-1.8(-3.4, -0.2)	0.03	0	-4.3(-8.8, 0.3)	0.07	0
NO_2 12-20	2322	-1.8(-3.4, -0.1)	0.02	0	-5.1(-12.7, 2.6)	0.19	56.6
NO_2 20-34	2223	-0.4(-2.2, 1.3)	0.61	27.6	-1.0(-8.6, 6.6)	0.79	51.5
**EFW size at 12**							
NO_2 0-12	2399	-1.6(-3.0, -0.3)	0.02	0	-1.2(-5.3, 3.0)	0.58	31.8
**EFW size at 20**							
NO_2 0-12	2208	-1.1(-2.6, 0.4)	0.14	0	0.2(-4.2, 4.5)	0.93	0
NO_2 12-20	2316	-0.4(-1.7, 0.9)	0.52	0	-3.4(-7.3, 0.6)	0.10	0
**EFW size at 34**							
NO_2 0-12	2317	-2.1(-3.7, -0.6)	0.01	0	-5.2(-9.6, -0.7)	0.02	0
NO_2 12-20	2316	-1.8(-3.3, -0.2)	0.03	0	-6.0(-13.6, 2.0)	0.14	59.1
NO_2 20-34	2254	-0.8(-2.5, 0.9)	0.34	0	-1.2(-9.5, 7.0)	0.77	60.4

Cohort-specific models for BPD were adjusted for rurality, alcohol consumption, energy intake, employment, and gestational weight gain. FL: rurality, vitamin D, energy intake, marital status, tobacco use, season, and gestational weight gain. AC: rurality, marital status, season, education, social class, employment, gestational weight gain, alcohol consumption, energy intake, and type

of cooking. EFW: rurality, season, GWG, alcohol consumption, energy intake, employment, tobacco use, type of cooking, and education. (a) % of difference in unconditioned SD scores obtained by combining cohort-specific estimates using meta-analysis. Note: Size at week 12 is the same as growth in 0-12 weeks, thus the estimates in this part completely match with those presented in table 2 under the label growth in 0-12 (b) p-value according to likelihood ratio (LR) test. (c) I^2 statistic of heterogeneity, estimated coefficient with $I^2 > 50\%$ were derived using random effects models.

Supplemental material, Table S3. Results of sensitivity analyses. INMA Study 2003-2008 (Spain)

	Confirmed LMP-based GA			≥ 15 hours/day spent at home			Term deliveries only			Common model		
**Outcome and Exposure**	**N**	**% diff (95% CI)[a]**	**p[b]**	**N**	**% diff (95% CI)[a]**	**p[b]**	**N**	**% diff (95% CI)[a]**	**p[b]**	**N**	**% diff (95% CI)[a]**	**p[b]**
**BPD growth: 0-12**												
NO_2 0-12	2093	-0.9(-2.3,0.6)	0.24	1429	-1.2 (-2.9, 0.5)	0.18	2277	-1.1 (-2.5, 0.2)	0.11	2253	-1.6 (-3.3, 0.0)	0.05*
**BPD growth: 12-20**												
NO_2 0-12	2024	-0.2 (-1.7, 1.3)	0.78	1391	-0.7 (-2.4, 1.0)	0.42	2215	-0.3 (-1.8, 1.2)	0.69	2253	-0.6 (-2.2, 1.1)	0.50
NO_2 12-20	2039	0.8 (-0.6, 2.2)	0.25	1400	0.6 (-1.0, 2.3)	0.45	2231	0.8 (-0.6, 2.1)	0.28	2254	-0.2 (-2.0, 1.6)	0.84
**BPD growth: 20-34**												
NO_2 0-12	2036	-2.7 (-4.1, -1.2)	<0.01*	1397	-2.1 (-5.3, 1.0)	0.19	2230	-3.1 (-4.4, -1.7)	<0.01*	2251	-2.2 (-3.8, -0.6)	0.01*
NO_2 12-20	2034	-1.9 (-3.3, 0.5)	0.01*	1394	-0.8 (-4.1, 2.4)	0.61	2227	-2.2 (-3.5, -0.8)	<0.01*	2252	-1.8 (-3.5, -0.2)	0.02*
NO_2 20-34	1948	-0.1 (-1.7, 1.5)	0.90	1362	-0.4 (-4.0, 3.3)	0.84	2140	-0.1 (-1.7, 1.4)	0.87	2251	-1.2 (-2.9, 0.5)	0.17
**BPD size at 34**												
NO_2 0-12	2040	-2.8 (-4.2, -1.3)	<0.01*	1399	-3.3 (-5.1, -1.6)	<0.01*	2233	-3.2 (-4.6, -1.8)	<0.01*	2255	-2.4 (-4.0, -0.7)	0.01*
NO_2 12-20	2038	-2.0 (-3.3, -0.6)	<0.01*	1398	-1.3 (-4.1, 1.6)	0.38	2232	-2.2 (-3.6, -0.9)	<0.01*	2253	-1.9 (-3.5, -0.3)	0.02*
NO_2 20-34	1985	-0.6 (-2.2, 1.0)	0.45	1395	-1.8 (-3.7, 0.1)	0.07	2182	-0.6 (-2.1, 0.9)	0.41	2251	-1.5 (-3.2, 0.3)	0.10
**FL growth: 0-12**												
NO_2 0-12	2025	-1.6(-3.2,-0.1)	0.03*	1373	-2.0 (-3.7, -0.2)	0.03*	2204	-1.6 (-3.1, -0.2)	0.03*	2250	-2.0 (-3.6, -0.3)	0.02*
**FL growth: 12-20**												
NO_2 0-12	2102	-0.2(-1.8,1.5)	0.83	1434	-1.3 (-3.3, 0.7)	0.20	2291	-0.4 (-2.0, 1.2)	0.63	2247	-0.8 (-2.5, 0.7)	0.31
NO_2 12-20	2105	-0.9(-2.3,0.5)	0.22	1438	-1.4 (-3.1, 0.3)	0.10	2294	-1.0 (-2.4, 0.3)	0.13	2250	-2.0 (-3.5, -0.4)	0.02*
**FL growth: 20-34**												
NO_2 0-12	1993	-0.6(-2.0,0.8)	0.40	1398	-0.8 (-2.5, 0.9)	0.37	2187	-0.9 (-2.3, 0.4)	0.18	2250	-0.4 (-2.1, 1.2)	0.59
NO_2 12-20	1978	0.2(-1.5,1.9)	0.83	1390	-0.2 (-2.2, 1.8)	0.81	2171	0.0 (-1.7, 1.6)	0.95	2250	0.1 (-1.5, 1.7)	0.89
NO_2 20-34	1991	-0.6 (-2.4,1.2)	0.52	1395	0.0 (-3.3, 3.3)	0.99	2183	-0.9 (-2.6, 0.8)	0.31	2247	-0.6 (-2.3, 0.0)	0.46
**FL size at 34**												
NO_2 0-12	2053	-0.9(-2.3,0.5)	0.23	1436	-1.0 (-2.7, 0.6)	0.21	2249	-1.2 (-2.5, 0.2)	0.08	2248	-0.7 (-2.3, 1.0)	0.42
NO_2 12-20	1991	0.3(-1.1,1.7)	0.67	1396	-0.1 (-1.7, 1.6)	0.91	2184	-0.2 (-1.5, 1.1)	0.78	2248	-0.4 (-2.0, 1.2)	0.65
NO_2 20-34	2050	-0.4(-1.9,1,0)	0.54	1434	-0.9 (-2.7, 0.8)	0.30	2245	-0.6 (-2.0, 0.8)	0.41	2245	-1.0 (-2.7, 0.6)	0.23
**AC growth: 0-12**												
NO_2 0-12	2101	-2.1(-3.7,-0.4)	0.01*	1432	-3.4 (-5.4, -1.4)	<0.01*	2290	-2.2 (-3.8, -0.6)	0.01*	2248	-2.4 (-4.0, -0.8)	<0.01*
**AC growth: 12-20**												
NO_2 0-12	1975	0.9(-0.8,2.6)	0.30	1389	1.3 (-0.8, 3.4)	0.22	2166	0.6 (-1.0, 2.3)	0.45	2249	0.8 (-0.9, 2.4)	0.35
NO_2 12-20	2034	1.0(-0.7,2.7)	0.23	1398	1.8 (-0.2, 3.8)	0.07	2225	0.8 (-0.8, 2.5)	0.30	2249	0.9 (-0.7, 2.5)	0.29

**AC growth: 20-34**												
NO_2 0-12	2033	-1.8(-3.5,-0.1)	0.04*	1396	-2.5 (-4.7, -0.4)	0.02*	2227	-2.2 (-3.9, -0.6)	0.01*	2252	-1.9 (-3.5, -0.2)	0.02*
NO_2 12-20	2034	-2.0(-3.7,-0.3)	0.02*	1397	-2.6 (-4.6, -0.5)	0.02*	2227	-2.1 (-3.7, -0.5)	0.01*	2253	-1.6 (-3.2, 0.0)	0.05*
NO_2 20-34	1880	-0.5(-2.4,1.4)	0.62	1309	-1.7 (-4.0, 0.7)	0.16	2063	-0.5 (-2.4, 1.3)	0.57	2251	-0.2 (-1.9, 1.5)	0.81
**AC size at 34**												
NO_2 0-12	2034	-1.8 (-3.5,-0.1)	0.04*	1397	-2.6 (-4.7, -0.5)	0.02*	2227	-2.3 (-3.9, -0.6)	0.01*	2253	-2.0 (-3.6, -0.3)	0.02*
NO_2 12-20	2033	-1.9(-3.6,-0.2)	0.03*	1397	-2.4 (-4.4, -0.3)	0.02*	2225	-2.2 (-3.9, -0.6)	0.01*	2251	-1.8 (-3.3, -0.2)	0.03*
NO_2 20-34	1949	-0.9(-2.7,1.0)	0.36	1363	-2.0 (-4.3, 0.3)	0.09	2140	-0.8 (-2.5, 1.0)	0.42	2251	-0.6 (-2.2, 1.2)	0.53
**EFW growth: 0-12**												
NO_2 0-12	2098	-1.8(-3.3,-0.4)	0.01*	1432	-2.5 (-4.2, -0.8)	<0.01*	2288	-1.9 (-3.3, -0.5)	0.01*	2244	-3.0 (-4.6, -1.3)	<0.01*
**EFW growth: 12-20**												
NO_2 0-12	1968	1.0(-0.7, 2.7)	0.26	1382	0.8 (-1.2, 2.9)	0.42	2161	0.7 (-1.0, 2.3)	0.42	2241	0.6 (-1.0, 2.2)	0.49
NO_2 12-20	1968	0.4(-1.4,2.0)	0.69	1382	0.7 (-1.3, 2.7)	0.49	2161	0.2 (-1.4, 1.8)	0.78	2242	0.4 (-1.4, 2.1)	0.68
**EFW growth: 20-34**												
NO_2 0-12	2020	-2.1(-3.7,-0.4)	0.02*	1387	-2.6 (-4.6, -0.5)	0.02*	2216	-2.6 (-4.2, -1.0)	<0.01*	2239	-2.0 (-3.7, -0.4)	0.01*
NO_2 12-20	2021	-1.7(-3.3,-0.0)	0.05*	1387	-2.6 (-4.7, -0.6)	0.01*	2216	-2.0 (-3.5, -0.4)	0.02*	2239	-1.2 (-2.8, 0.4)	0.13
NO_2 20-34	1974	-0.5(-2.3,1.3)	0.59	1387	-1.6 (-3.8, 0.6)	0.16	2169	-0.6 (-2.4, 1.1)	0.46	2240	-0.3 (-2.0, 1.4)	0.71
**EFW size at 34**												
NO_2 0-12	2027	-2.0(-3.7,-0.3)	0.02*	1394	-2.8 (-4.8, -0.6)	0.01*	2223	-2.6 (-4.2, -1.0)	<0.01*	2243	-2.1 (-3.7, -0.5)	0.01*
NO_2 12-20	2026	-1.9(-3.5,-0.2)	0.03*	1393	-2.4 (-4.3, -0.3)	0.02*	2222	-2.2 (-3.8, -0.6)	0.01*	2242	-1.6 (-3.2, 0.0)	0.05*
NO_2 20-34	1976	-1.2(-2.9,0.6)	0.20	1389	-1.8 (-3.9, 0.4)	0.10	2170	-1.0 (-2.8, 0.6)	0.23	2239	-1.1 (-2.8, 0.6)	0.22

(a) % of difference in SD scores. (b) p-value according to LR-test, *$p<0.05$. Effect estimates per 10 $\mu g/m^3$ increase in NO_2.

Supplemental Material, Figure S7. Shape of the relationship between exposure to NO_2 in weeks 0-12 and size of fetal parameters at week 12. INMA Study 2003-2008 (Spain)

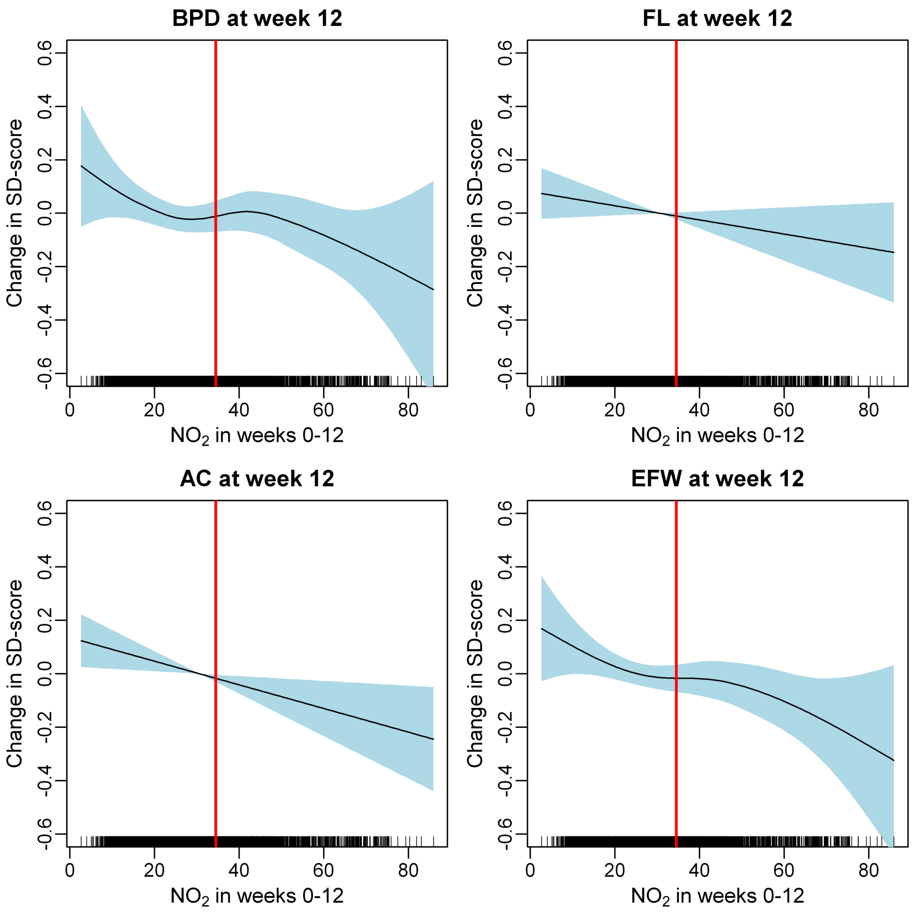

Shape estimated from a generalized additive model using a penalized cubic spline as a smoother. According to AIC, linearity was rejected for BPD and EFW, and the same pattern could be appreciated in these two cases. The vertical line is plotted at the 66th percentile of the distribution of NO_2 levels.

Supplemental Material, Figure S8. Shape of the relationship between exposure in the critical window of NO_2 (weeks 0-12 or weeks 12-20) and size of fetal parameters at week 20. INMA Study 2003-2008 (Spain)

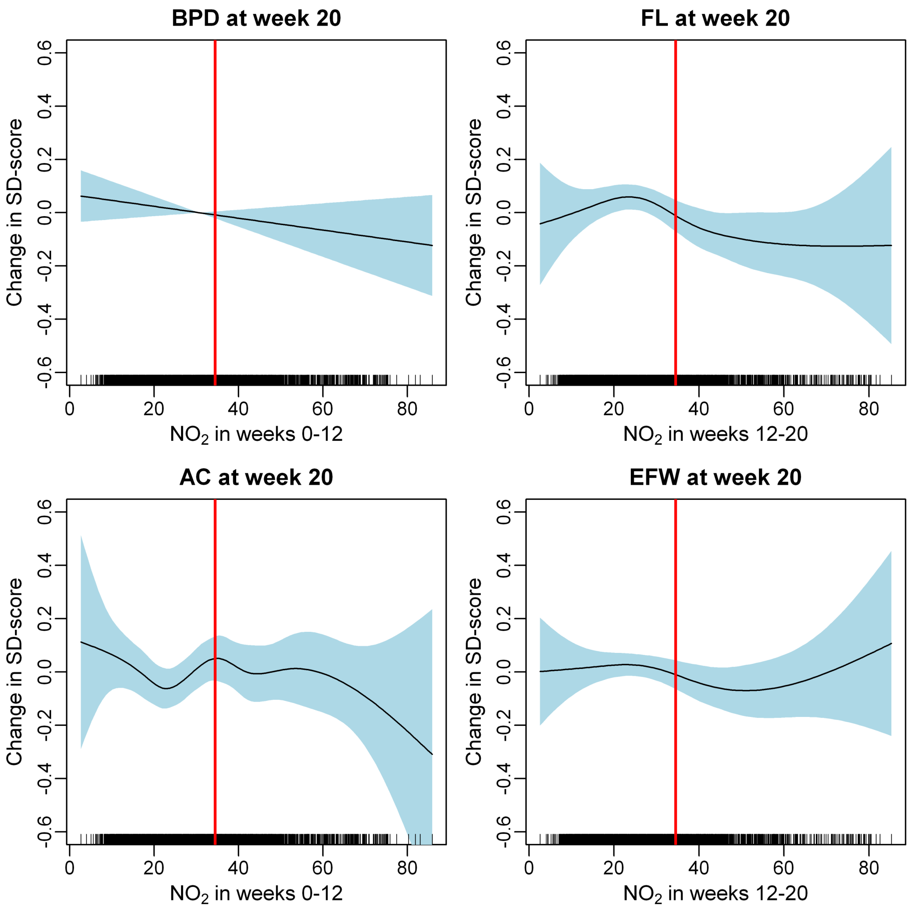

According to AIC, linearity was rejected for FL and EFW, and the same pattern could be appreciated in these two cases.

Supplemental Material, Figure S9. Shape of the relationship between exposure in the critical window of NO_2 (weeks 0-12) and size of fetal parameters at week 34. INMA Study 2003-2008 (Spain)

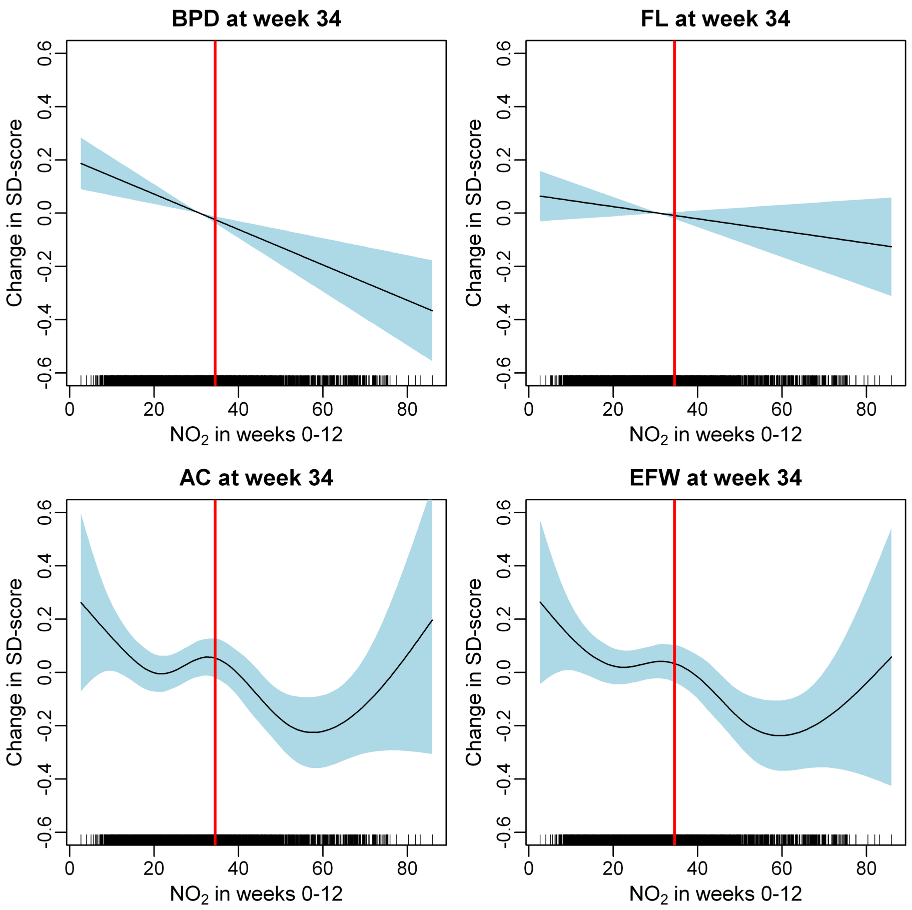

According to AIC, linearity was rejected for AC and EFW, and the same pattern could be appreciated in these two cases.

References

Gurrin LC, Blake KV, Evans SF, Newnham JP. 2001. Statistical measures of foetal growth using linear mixed models applied to the foetal origins hypothesis. Stat Med 20:3391-3409.

Mamelle N, Cochet V, Claris O. 2001. Definition of fetal growth restriction according to constitutional growth potential. Biol Neonate 80:277-285.

Pinheiro JC, Bates DM. 2000. Mixed-effects models in S and S PLUS. Statistics and computing. New York:Springer-Verlag Inc.

Royston P, Altman DG. 1994. Regression Using Fractional Polynomials of Continuous Covariates: Parsimonious Parametric Modelling. Applied Statistics 43:429-467.

Royston P. 1995. Calculation of unconditional and conditional reference intervals for foetal size and growth from longitudinal measurements. Stat Med 14:1417-1436.
